# Efficacy and Safety of Pharmacological and Physical Therapies for Bell's Palsy: A Bayesian Network Meta-Analysis

**DOI:** 10.3389/fneur.2022.868121

**Published:** 2022-04-18

**Authors:** Jianwei Shi, Dafeng Lu, Hairong Chen, Mingzhu Shu, Yang Xu, Jiaojiao Qian, Ke Ouyang, Huaying Huang, Zhengxiang Luo, Chunhui Wang, Yansong Zhang

**Affiliations:** ^1^Department of Neurosurgery, The Affiliated Brain Hospital of Nanjing Medical University, Nanjing, China; ^2^School of Public Health, Nanjing Medical University, Nanjing, China; ^3^Department of Neurology, The Affiliated Brain Hospital of Nanjing Medical University, Nanjing, China; ^4^Department of Infectious Diseases, Jiangsu People's Hospital and Nanjing Medical University First Affiliated Hospital, Nanjing, China; ^5^Center for Disease Control and Prevention of Eastern Theater Command, Nanjing, China

**Keywords:** Bell's palsy, pharmacological therapy, physical therapy, network meta-analysis, systematic review

## Abstract

**Objective:**

The objective was to comprehensively assess the efficacy and safety of all pharmacological and physical treatments (short-term, ≤ 1 month) for patients with acute Bell's palsy.

**Methods:**

The electronic databases PubMed, Web of Science, Embase, Cochrane Library, and CNKI were searched for the randomized controlled trials comparing two or more regimens in patients with the Bell's palsy to be included in a Bayesian network meta-analysis. Odds ratios and CIs for the primary outcome of the House–Brackmann scale and secondary outcomes of sequelae (synkinesis and crocodile tears) and adverse events were obtained and subgroup analyses of steroids and antivirals were conducted.

**Results:**

A total of 26 studies representing 3,609 patients having undergone 15 treatments matched our eligibility criteria. For facial recovery, acupuncture plus electrical stimulation, steroid plus antiviral plus Kabat treatment, and steroid plus antiviral plus electrical stimulation were the top three options based on analysis of the treatment ranking (probability = 84, 80, and 77%, respectively). Steroid plus antiviral plus electrical stimulation had the lowest rate of sequelae but were more likely to lead to mild adverse events. Subgroup analysis revealed that methylprednisolone and acyclovir were likely to be the preferred option.

**Conclusions:**

This network meta-analysis indicated that combined therapies, especially steroid plus antiviral plus Kabat treatment, were associated with a better facial function recovery outcome than single therapy. Other physical therapies, such as acupuncture plus electrical stimulation, may be a good alternative for people with systemic disease or allergies. More high-quality trials of physical regimens are needed in the future.

**Systematic Review Registration:**

Our registered PROSPERO number is CRD42021275486 and detailed information can be found at https://www.crd.york.ac.uk/PROSPERO/.

## Introduction

Bell's palsy, also known as idiopathic facial nerve palsy, is an acute, unilateral facial paresis or paralysis of unknown origin, and is the most common cause of lower motor neuron facial palsy (1), with an annual incidence rate ranging from 11.5 to 53.3 per 1,00,000 (2) and can occur at any age with a lifetime risk of 1 in 60 (3). The precise cause for Bell's palsy remains unclear, yet the reactivated latent herpes simplex virus type 1 (HSV-1) infection has been widely proposed as its major cause (4–6). Due to facial nerve dysfunction, patients with Bell's palsy are frequently unable to move facial expression muscles voluntarily on the affected side, presenting also with postauricular pain, dysgeusia, hyperacusis, and dryness of the eye or mouth (2, 5). Although 70% of patients can recover completely within 6 months even without treatment, up to 30% of patients fail to fully recover, and even half of them will suffer moderate-to-severe sequelae, such as hemifacial spasm, crocodile tears (abnormal lacrimation with eating), contracture and synkinesis (3, 7). Therefore, the effective and safe improvement of the recovery rate in Bell's palsy is of great significance.

Currently, the treatments for Bell's palsy can be categorized into two strategies, namely, pharmacotherapy and physiotherapy, both aimed at facial function recovery facilitation and sequelae prevention (8–10). In the majority of the cases, pharmacological therapy is preferred in the early stage of Bell's palsy, including corticosteroids and antiviral agents, either individually or in combination (9–12). Corticosteroids are the most commonly used drugs for Bell's palsy due to their potent anti-inflammatory effect, which can reduce edema and inflammation of the facial nerve. Available evidence from randomized controlled trials (RCTs) has revealed benefits from treating the Bell's palsy with corticosteroids (9, 13). Many published authoritative clinical practice guidelines for Bell's palsy have strongly recommended the prescription of corticosteroids within 72 h of symptom onset (2, 14, 15). However, the benefit of antiviral agents has not yet been established, and antiviral therapy alone is discouraged in the treatment of Bell's palsy (2, 10, 14, 15). As for combined antiviral and corticosteroid treatment, it remains a matter of extensive debate with conflicting conclusions emerging from different meta-analyses (5, 10, 16). On the other hand, physical therapy can be beneficial and complementary from even alternative therapies to pharmacological therapy. Thermotherapy, massage, electrostimulation, facial exercises, Kabat rehabilitation, and acupuncture are regarded as different forms of physical therapy that have been widely applied for Bell's palsy in the clinical setting (2, 8). Although many studies have suggested that physical therapy has a good therapeutic effect on Bell's palsy with few side effects, the lack of high-quality RCTs limits verification of its efficacy and risk (8, 17).

In clinical practice, patients with Bell's palsy tend to be treated with various treatment options in different departments. Controversy remains regarding the optimal treatment protocol for these patients. Previous systematic reviews and meta-analyses have been conducted to compare pharmacological therapies and physical therapies, respectively (8, 12, 17, 18). Nevertheless, no study has been performed so far that compares all available interventions for the management of Bell's palsy, including pharmacological and physical therapies, which may be beneficial in obtaining the best treatment. In this study, a network meta-analysis (NMA) was conducted, which integrated data from direct and indirect comparisons of various current therapies, to comprehensively compare the efficacy and safety of pharmacological therapies and physical therapies for the treatment of Bell's palsy.

## Methods

### Study Design and Search Strategy

The present meta-analysis was conducted following the recommendations of the Preferred Reporting Items for Systematic Reviews and Meta-Analyses (PRISMA) for NMA guidelines, and our protocol was registered in the International prospective register of systematic reviews—PROSPERO (CRD42021275486) (19). A computerized search was performed for studies published on PubMed, Web of Science, Embase, Cochrane Library, and CNKI from 1990 to 25 October 2021, using the following Medical Subject Headings (MeSH) terms and keywords: “Bell's palsy”, “facial neuritis”, “facial paralysis”, “therapeutics”, “drugs”, “acupuncture therapy”, “hormones”, and “antiviral agents.” Only randomized trials (RCTs) were included in our study. A total of three investigators (J. Shi, D. Lu, and M. Shu) screened the reference lists of included studies, relevant reviews, and other relevant documents to identify additional studies not included in the initial search.

### Study Selection

Study selection was conducted following a pre-set eligibility criteria set by J. Shi and D. Lu. Inclusions were as follows: (1) Patients who were treated for acute and unilateral facial paralysis of unknown cause. (2) Trials in which at least one treatment was compared with a placebo. (3) Trials in which any two or more different routes of physical or pharmacological treatments were compared. (4) Reporting recovery results of facial function, which could be measured using the House–Brackmann scale (HBS). Other exclusion criteria were as follows: (1) Face paralysis caused by other diseases. (2) More than 14 days from onset of palsy to start of treatment. (3) Studies with insufficient data. Studies not adhering to the inclusion criteria were excluded.

### Data Extraction

Data on trial details (e.g., first author, publication year, implementation country, number of patients, and patient characteristics), treatments, and outcomes were independently extracted by two reviewers (J. Shi and D. Lu), and then censored by another reviewer (J. Qian). Only treatment-related adverse events were used in the analysis. If multiple follow-up periods existed in one study, only data on the final follow-up period were extracted. Unavailable information of any of the aforementioned categories was documented as NR (not reported). The quality and risk of bias of the selected RCTs were assessed using the revised Cochrane risk-of-bias tool for randomized trials according to different outcomes. Items were considered as low, high, or uncertain risks of bias. Any discrepancies were resolved by consensus and arbitration by the authors.

### Statistical Analysis

Evidence to compare different treatments in terms of efficacy and safety was synthesized and these parameters were reported as odds ratios (ORs) and 95% credible interval (CI) for each study. The primary endpoint was the ORs for facial function recovery according to HBS. Secondary outcomes were the ORs for sequelae (synkinesis and crocodile tears) and adverse events.

Fixed-effects, pairwise meta-analysis within a frequentist framework was performed on head-to-head comparisons. Heterogeneity between studies was assessed using the *Q* test and *I*^2^ statistic within a visual forest plot. The statistical significance level was set to *P* ≤ 0.05. Low, moderate, or high heterogeneity levels were considered for the estimated *I*^2^ values under 25%, between 25 and 50%, and over 50%, respectively. The global inconsistency was evaluated by comparing the fit of consistency and inconsistency models, where *P* ≤ 0.05 indicated inconsistency.

A randomized effects consistency model was used. The model fit of each analysis was assessed by the deviance information criterion (DIC) (20). For HBS, sequelae, and adverse events, 50,000 sample iterations were generated with 20,000 burn-ins and a thinning interval of one. The convergence of iterations was evaluated by visual inspection of the four chains to establish homogenous parameter estimates in accordance with the Brooks–Gelman–Rubin diagnostic. Once convergence was established, the posterior distributions for the model parameters were obtained as the output of the NMA estimate (OR and the corresponding 95% CI). In the presence of minimally informative priors, credible intervals can be interpreted such as conventional confidence intervals. NMA estimated the overall rankings of treatments by calculating the surface under the cumulative ranking curve (SUCRA) for each, which approaches one when a treatment is certain to be the best and zero when treatment is the worst (21).

All analyses were conducted using the gemtc2 and netmeta package of R (version 4.1.1) to generate network plots to illustrate the geometries, to clarify which treatments were compared directly or indirectly in the included studies.

### Meta-Regression Analysis

In general studies, follow-up time, age, sex, and some other factors may interfere with the results of facial recovery. Therefore, we investigated whether these covariates, as well as treatment factors, were related to changes in facial recovery. Using the metafor package (version 2.0.0), we did meta-regression using placebo-controlled data aiming to examine the relationship between facial functional changes after treatments and baseline HBS, baseline level of a given parameter, age, and sex. In these meta-regressions, if a study had multiple active groups, estimates for each group were merged.

### Risk of Bias Across Studies

The Grading of Recommendations, Assessment, Development, and Evaluation (GRADE) assessment was performed by J. Shi and D. Lu and discussed with the rest of the authors. Using the GRADE approach, the overall quality of clinical recommendations (confidence in effect estimates) derived from NMA results was assessed (19). The GRADE system evaluates the quality of evidence at four levels: high, moderate, low, and very low with rating aspects of (a) study limitations, (b) inconsistency, (c) indirectness, (d) imprecision, and (e) publication bias.

## Results

### Description of the Studies

A total of 26 studies were identified for analysis at the full-text screening stage; detailed reasons are presented in Supplementary Figure 1. These studies, published from 1993 to 2020 and conducted in 12 countries, had a total of 3,609 patients enrolled that received 15 different treatments, which are presented in Supplementary Table1.

### Network Meta-Analysis in Treatments for Bell's Palsy

**Figure 1:**
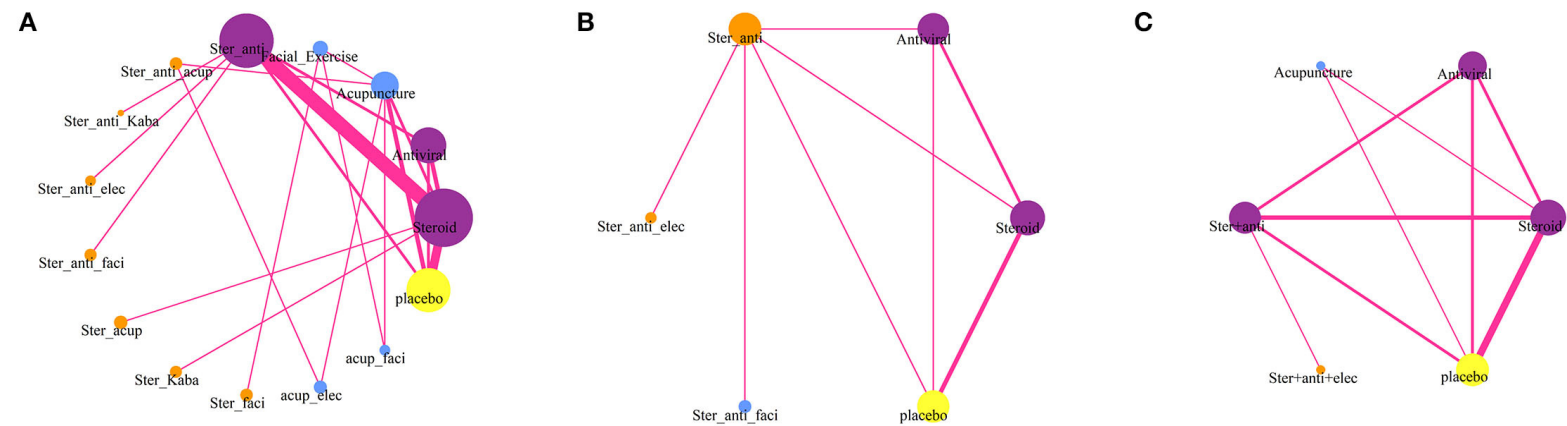
Network diagrams of comparisons on different outcomes of treatments of patients with Bell's palsy. **(A)** Comparisons on facial recovery in patients with Bell's palsy. **(B)** Comparisons on sequelae in patients with Bell's palsy. **(C)** Comparisons on adverse events in patients with Bell's palsy. The node size is proportional to the total number of patients receiving treatment. Each line represents a type of head-to-head comparison. The width of lines is proportional to the number of trials comparing the connected treatments (Ster, Steroid; Anti, antiviral; Kaba, Kabat treatment; Elec, Electrical stimulation; Acup, Acupuncture; Faci, Facial exercise).

**Figure 2:**
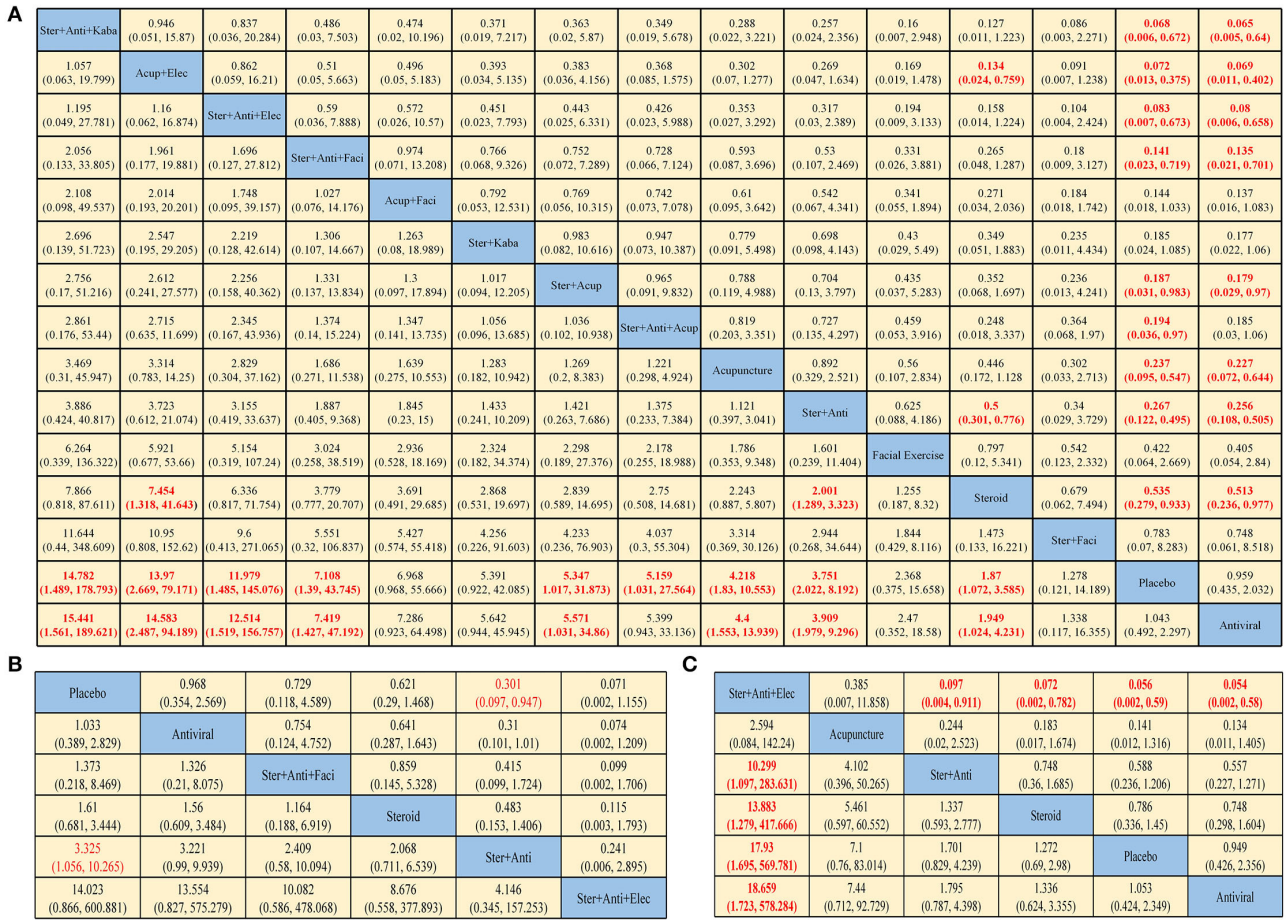
Pooled estimates of the network meta-analysis. Pooled hazard ratios (95% credible intervals) for facial recovery **(A)**, sequelae **(B)**, and adverse events **(C)**. Data in each cell are odds ratios (95% credible intervals) for the comparison of row-defining treatment vs. column-defining treatment. Odds ratios more than 1 favor row-defining treatment. Significant results are in red (Ster, Steroid; Anti, antiviral; Kaba, Kabat treatment; Elec, Electrical stimulation; Acup, Acupuncture; Faci, Facial exercise).

The network meta-analysis included data of 15 treatments for recovery of HBS (Figure 1A), but only six treatments had related data for sequelae (Figure 1B) and adverse events (Figure 1C). The results providing indirect comparisons between treatments are presented in Figure 2.

In terms of HBS (Figure 2A), steroid plus antiviral plus Kabat treatment yielded the highest benefit of HBS recovery vs. placebo (OR 15.441, 95% CI: 1.561–189.621), followed by acupuncture plus electrical stimulation (OR 14.583, 2.487–94.159), steroid plus antiviral plus electrical stimulation (OR 12.514, 1.519–156.757) and steroid plus antiviral plus facial exercise (OR 7.419, 1.427–47.192). The use of just antivirals seems to have limited efficacy.

In terms of sequelae (Figure 2B), the difference between the interventions is not obvious. Only steroid plus antiviral was statistically significantly effective in preventing sequelae, compared with placebo (OR 3.325, 1.056–10.265). Steroid plus antiviral plus electrical stimulation has a higher rate of causing adverse events compared with other treatments (Figure 2C). Dyspepsia, nausea, vomiting, diarrhea, and constipation are relatively common symptoms in treatments with corticosteroids, while pain, contact dermatitis, and ecchymosis are often seen in physiotherapy.

### Rank Probabilities

**Figure 3:**
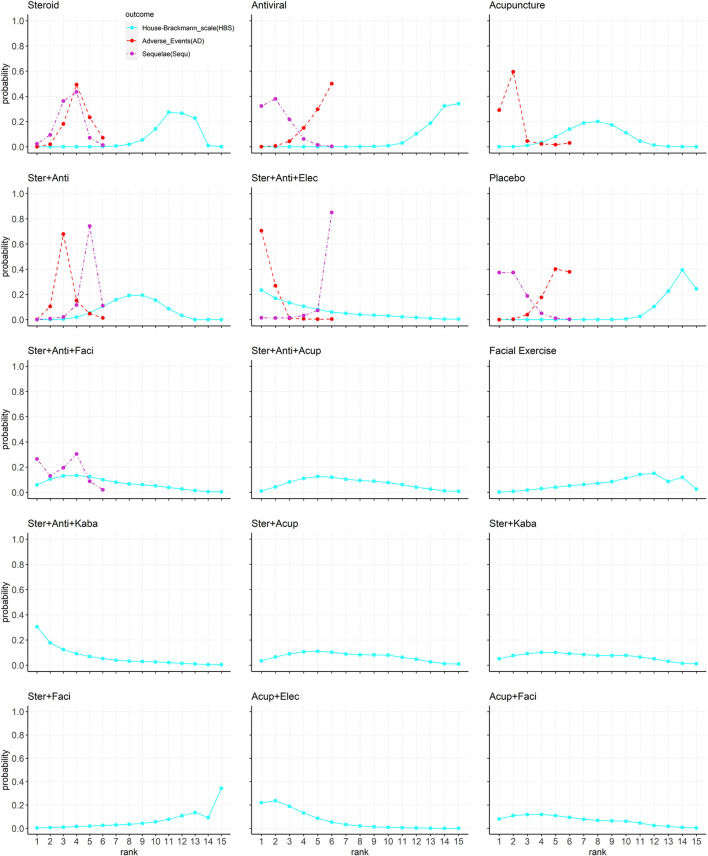
The Bayesian ranking profiles of comparable treatments on efficacy for patients with Bell's palsy according to House–Brackmann rating scale. Profiles indicate the probability of 15 comparable treatments being ranked from first to last on facial recovery (blue), adverse events (red), and sequelae (purple). Ranking curves are described according to the Bayesian ranking results presented in Supplementary Table 2 (Ster, Steroid; Anti, antiviral; Kaba, Kabat treatment; Elec, Electrical stimulation; Acup, Acupuncture; Faci, Facial exercise).

The Bayesian ranking profiles of comparable treatments are shown in Figure 3 and Supplementary Table 2. The Bayesian ranking results are almost in line with the pooled analyses using odds ratios. The SUCRA plots for patients with Bell's palsy show that acupuncture plus electrical stimulation ranked first for facial function recovery (cumulative probability: 84%), followed by steroid plus antiviral plus Kabat treatment (80%) and steroid plus antiviral plus electrical stimulation (77%). Steroid plus antiviral plus electrical stimulation was most likely to cause adverse events (93%), followed by acupuncture (81%). Antiviral had the lowest probability (15%) of causing adverse events. Placebo was most likely to cause sequelae (81%), and steroid plus antiviral plus electrical stimulation had the lowest probability (6%) to cause sequelae.

### Explore of Potential Interaction

**Figure 4:**
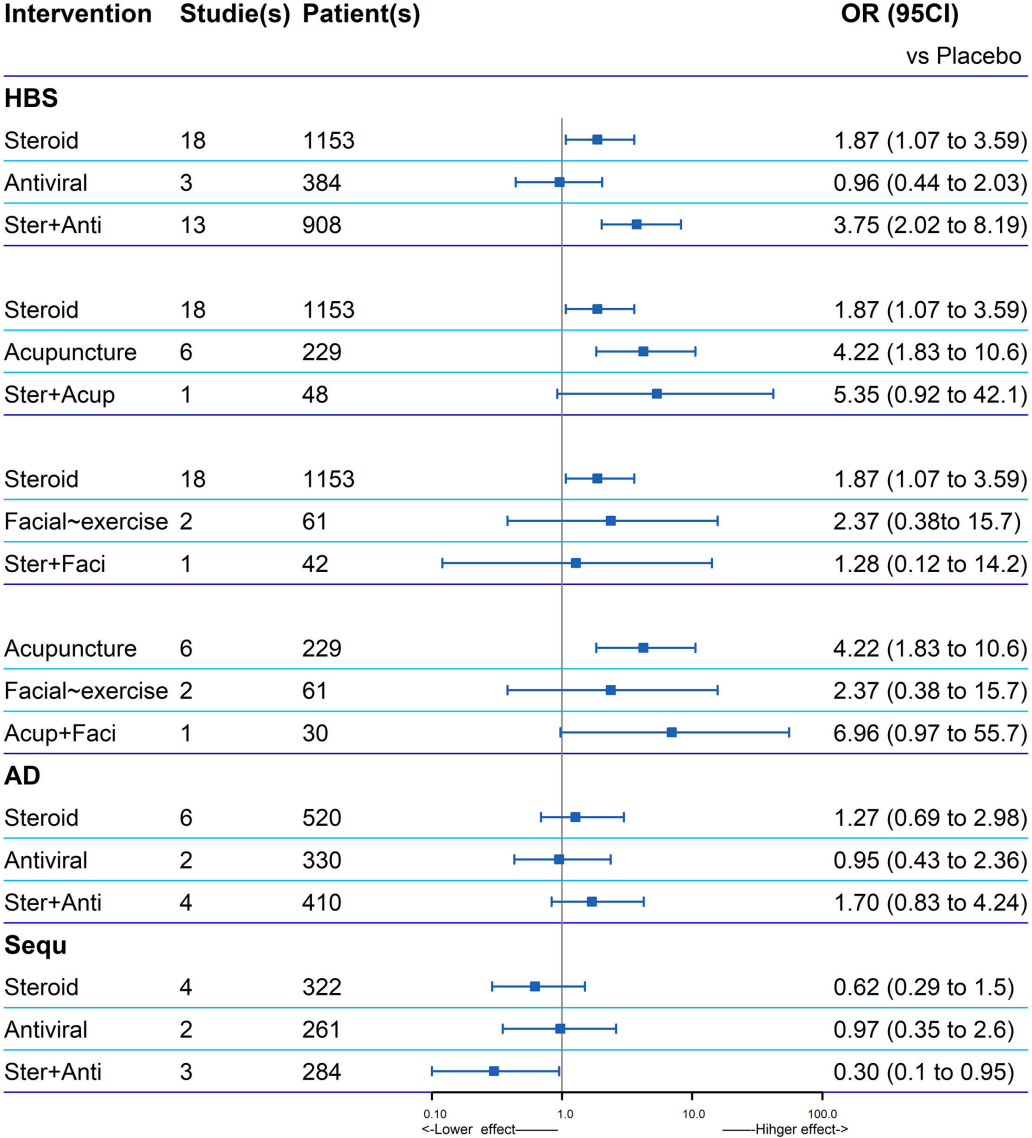
Explore of potential interaction of four combined treatments and their corresponding single treatment for the facial recovery. If one is not contained in the 95% CI of the combination therapy, the two single treatments in the combination therapy are considered to have a positive interaction significantly (HBS, House–Brackmann scale; AD, adverse events; Sequ, sequelae; Ster, Steroid; Anti, antiviral; Acup, Acupuncture; Faci, Facial exercise).

The data of four combined treatments and their corresponding single treatment can be extracted and analyzed (Figure 4). According to the recovery of HBS, the OR value of the combined treatments compared with placebo was higher than the related single treatment, except for steroid plus facial exercise (OR: 1.28, 0.12–14.2). Steroid and antiviral treatment had a potential positive interaction both in facial function recovery (OR: 3.75, 2.02–8.19) and reducing sequelae (OR: 0.3, 0.10–0.95).

### Subgroup Analysis

**Figure 5:**
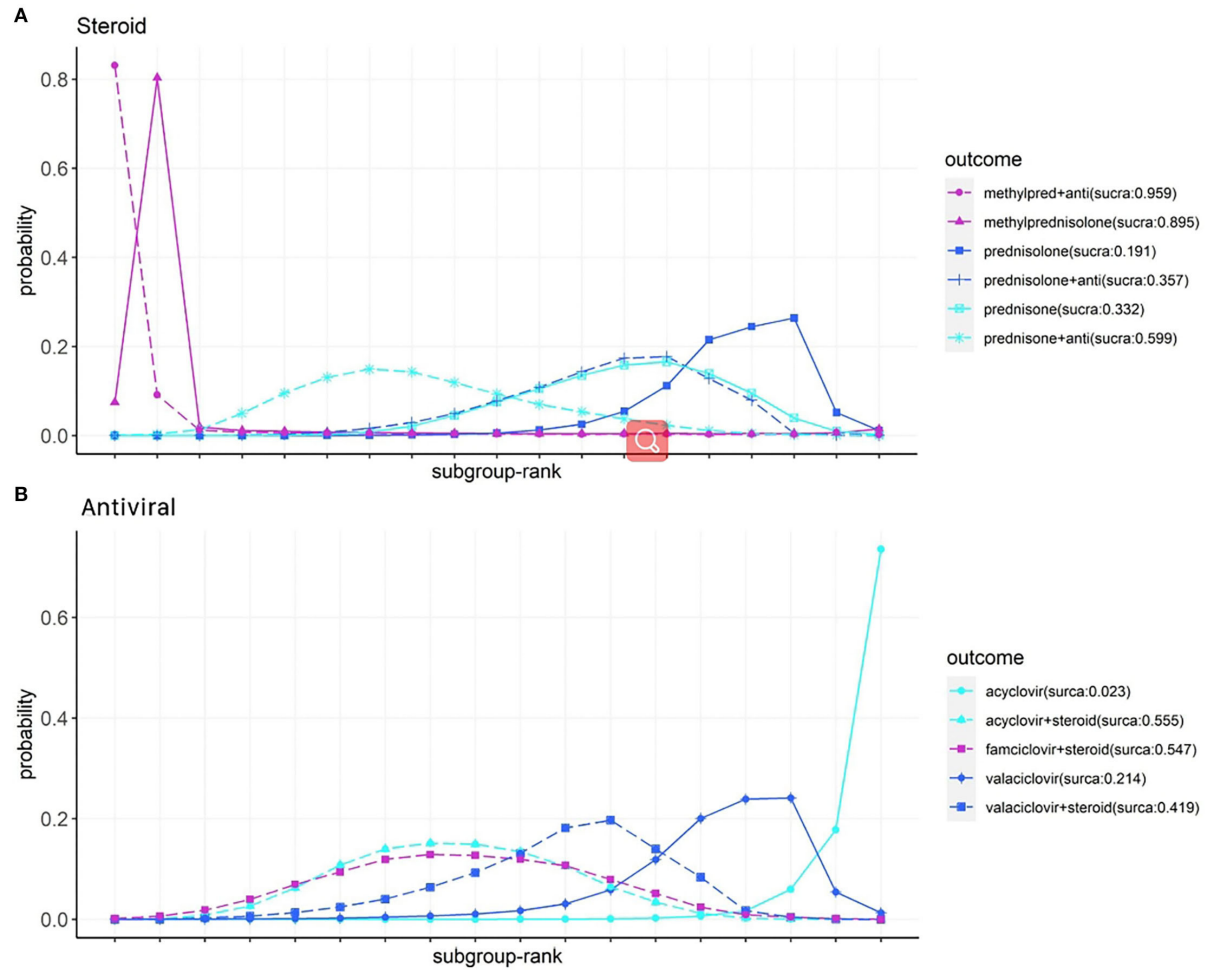
Subgroup analysis of steroids **(A)** and antivirals **(B)** on facial recovery for patients with Bell's palsy, according to Bayesian ranking profiles. Profiles indicate the probability of each comparable treatment being ranked from first to last on facial recovery (Anti, antiviral; methyl pred, methylprednisolone).

The ranking of the various steroid and antiviral treatments according to the recovery of HBS is illustrated in Figure 5, based on two new formatted networks (Supplementary Figure 7). Analysis of the SUCRA plots reveals that in the steroid group (Figure 5A), methylprednisolone plus antiviral had the best results (96%), followed by methylprednisolone (90%) and prednisone plus antiviral (60%). In the antiviral group (Figure 5B), acyclovir plus steroid had the best results (56%), followed by famciclovir plus steroid (55%) and valaciclovir plus steroid (42%).

### Study Control

The methodological quality of the 26 studies is summarized in Supplementary Table 3. A significance level of *P* > 0.05 was noted for all cases, which indicated that inconsistency was not present in any comparison. All details and original data of testing inconsistency, group heterogeneity, and publication bias are attached to the Supplementary Files. We did not find strong evidence of an association between change in follow-up time (*k* = 26, *z* = 0.020, estimate = 0.006, *p* = 0.84), age (*k* = 26, estimate = −0.016, *z* =−1.38, *p* = 0.17), sex (*k* = 26, estimate = −0.009, *z* = −0.75, *p* = 0.45), publication year (*k* = 26, estimate = 0.013, *z* = 0.99, *p* = 0.32), and countries (*k* = 26, estimate = 0.011, *z* = 0.06, *p* = 0.96) with any baseline variables according to the regression test (Supplementary Table 4), which indicated that these factors would not influence the comparison results between various therapies. Upon pairwise comparison of multiple treatments, a ranking on efficacy and safety for those reported in the RCT studies can be created. The certainty of the evidence was evaluated using the GRADE system.

## Discussion

### Main Findings and Interpretation in the Context of Previous Research

The NMA conducted in this study comprehensively summarized the comparative efficacy and safety of 15 currently available pharmacological and physical therapies for patients with Bell's palsy, including 3,609 patients randomly assigned to corticosteroid, antiviral, acupuncture, electrical stimulation, Kabat treatment, facial exercise, and combined therapy or placebo treatment. This study also has the largest number of included patients and therapies for Bell's palsy to the knowledge of the authors. Our results suggest that:

* Combined therapy seemed to be more effective than single therapy, according to the facial function recovery. Steroid plus antiviral plus Kabat treatment, steroid plus antiviral plus electrical stimulation, and acupuncture plus electrical stimulation were the top three promising treatments for Bell's palsy. The efficacy of just antiviral treatment seemed to have limited efficacy.
* Steroid plus antiviral plus electrical stimulation had the lowest rate of sequelae but was more likely to cause mild adverse events. Steroid plus antiviral was statistically significantly effective in preventing sequelae.
* In subgroup analysis for combined treatment of steroid plus antiviral, methylprednisolone and acyclovir showed an advantage regarding the facial function recovery.

Pharmacotherapy represented by corticosteroids remains the most commonly used regimen for Bell's palsy. Corticosteroids can reduce oedema and inflammation of the facial nerve due to their anti-inflammatory mode of action. Antivirals have been used in clinical practice since the isolation of the herpes simplex virus-1 genome from the facial nerve endoneurial fluid of people with Bell's palsy (4, 22). Pairwise meta-analyses have also been conducted to evaluate the efficacies of different pharmacotherapies for facial function recovery (9, 18, 23). In present work, steroid plus antiviral was found to be substantially superior to steroid (OR: 2.00, 1.29–3.32), antiviral (OR: 3.91, 1.98–9.30) and placebo (OR: 3.75, 2.02–8.19) treatment, in terms of facial function recovery. Steroid and antiviral treatment have a positive interaction (OR: 2.09, 1.13–4.58) of facial function recovery, compared with placebo. Antiviral treatment alone had the highest probability (34%) of ranking the last in facial function recovery, followed by placebo (24%). Adverse events and the severity of the sequelae (synkinesis and crocodile tears) may determine the prognosis of facial palsy, and the severity of synkinesis did not appear to be always proportional to the degree of the residual paralysis of voluntary movement (9, 24). No significant difference was found in adverse events or motor synkinesis during follow-up of different pharmacotherapies. However, steroid and antiviral treatment were noted as a potential positive interaction to reduce the instances of long-term sequelae (OR: 0.3, 0.1–0.95), including motor synkinesis and crocodile tears, which was consistent with the findings of this study (25).

In the subgroup analysis of pharmacotherapies for facial function recovery, methylprednisolone alone or methylprednisolone plus antivirals showed good efficacy (cumulative probability: 96 and 90%, respectively). However, due to the limited number of studies and sample size, the CI of the results was wide, lowering the reliability of the results. The reported research and NMAs in treating classical Bell's palsy with drugs remain controversial (5, 23, 26). In our analysis, steroids with acyclovir, famciclovir, and valaciclovir have a similar cumulative probability (56, 55, and 42%). However, a single antiviral drug seemed to be less effective.

Physiotherapy is a potentially good alternative for people with systemic disease or allergies (8, 27, 28). Kabat treatment, also known as proprioceptive neuromuscular facilitation, consists of the facilitation of the voluntary response of an impaired muscle through the global pattern of an entire muscular section that undergoes resistance (29). Kabat rehabilitation is particularly indicated to prevent or treat synkinesis due to its focus on motor control of facial movements triggered by different feedback stimulation (29). Electrical stimulation may improve the facial function of patients with Bell's palsy by using transcutaneously delivered low amplitude, pulsed, electrical current to activate motor nerves innervating weak muscles (30). However, the genuine role that electrostimulation plays in Bell's palsy treatment is still controversial due to unstandardized parameters of frequency, intensity, pulse duration, treatment time, and a number of contractions. Facial exercise is an active motion exercise that consists of different facial muscles/motions with variations of speed, amplitude, and force to promote motor control and to avoid altered patterns of movements and overactivity of the unaffected side (31). The individualization of facial neuromuscular exercise is really important. By inserting needles into specific body regions (acupoints), acupuncture works to stimulate reflexes that activate peripheral nerves and transmit sensory information from the spinal cord to the brain, thus modulating our body physiology (27, 32). Evidence on acupuncture therapies is still underused in clinical practice.

Trials of any form of physical therapy were included to be compared with either drugs or an alternative form of non-drug treatment. Studies adding physical therapy (i.e., Kabat treatment, facial exercises, acupuncture, and electrical stimulation) to pharmacotherapy showed evidence of additional benefit. Liu et al. (33) introduced the mechanism of how electroacupuncture drives the anti-inflammatory vagal–adrenal axis. Many studies have claimed that the curative effect of electroacupuncture on Bell's palsy is significant (34). Our results showed that acupuncture plus electrical stimulation, or electroacupuncture, showed significant efficacy compared with steroids (OR: 7.45, 1.32–41.64), placebo (OR: 13.97, 2.97–79.17), and antivirals (OR: 14.58, 2.49–94.19). Steroid plus antiviral plus Kabat treatment and steroid plus antiviral plus electrical stimulation ranked in the top 2 for facial function recovery. The adverse events of physiotherapy, especially electrical stimulation, and acupuncture, were mostly pain, contact dermatitis, and ecchymosis. The commonly used acupoints for Bell's palsy are summarized in Supplementary Table 4.

### Strength and Limitations

First, this study established comparisons among all monotherapies and combination treatments for patients with Bell's palsy, and comprehensively analyzed the major efficacy, toxicity outcomes, and sequelae. Second, potential interactions can be discussed due to the large number of combination therapies included in the study. Both direct comparison (heterogeneity within groups) and indirect comparison (inconsistency between groups) are sufficient. Finally, the heterogeneity was relatively low in our analysis, so a subgroup analysis may be not necessary, but a subgroup analysis of drugs was still conducted to make our results more comprehensive.

Although this study used the largest number of included studies and participants compared with relevant studies, the additional studies did not contribute to the certainty of the evidence. Many included treatments were compared indirectly, and much direct evidence was from the same trial in the present network. Though the results of meta-regression showed that follow-up time, as well as some other factors, did not increase or decrease the OR between intervention and control groups, the follow-up of included studies was still relatively short with four studies reporting a follow-up as ≤ 2 months, which may cause the heterogeneity of results of facial recovery to some extent. In addition, the interpretation of the results was limited because the external validity of some evidence was relatively low. The SUCRA findings can be inaccurate, especially in our subgroup analysis. Finally, the limited treatments in subgroup analysis made evaluation of all the corticosteroids or antivirals included in our analysis impossible and may lead to potential publication bias as well.

### Suggestions for Future Research

First, if possible, long-term and high-quality RCTs studies, are required for a profound understanding of the benefits of different regimens, especially physiotherapy, for Bell's palsy. Second, trials that examine the recovery rate of Bell's palsy by gender or age are also required to investigate the effect of gender and aging on the study outcome.

## Conclusion

This study is considerably more comprehensive than previous meta-analyses on Bell's palsy due to its exhaustive search of pharmacological and physical therapies. Combined therapy especially steroid plus antiviral plus Kabat treatment is associated with a better facial function recovery outcome than single therapy. The effect of physical therapy is present, but is usually combined with other drugs, and further trials are needed to provide long-term follow-up data for a complete assessment. Steroid plus antiviral plus electrical stimulation treatment had the lowest rate of sequelae. Nevertheless, this research still lacks adequate results of adverse effects and sequelae. Further studies should include patient's important outcomes such as sequelae (crocodile tears and synkinesis), adverse events, and quality of daily life, using clearer random methods.

## Data Availability Statement

The original contributions presented in the study are included in the article/Supplementary Material, further inquiries can be directed to the corresponding author/s.

## Author Contributions

JS, DL, CW, and YZ: conception and design. JS, DL, HC, and YZ: methodology. JS, HC, KO, HH, and JQ: investigation. JS, DL, MS, and JQ: formal analysis. JS and DL: writing original draft. JS, ZL, CW, and YZ: writing—review and editing. ZL, CW, and YZ: supervision. All authors had full access to the data in the study and take responsibility for the integrity of the data and the accuracy of the data analysis.

## Conflict of Interest

The authors declare that the research was conducted in the absence of any commercial or financial relationships that could be construed as a potential conflict of interest.

## Publisher's Note

All claims expressed in this article are solely those of the authors and do not necessarily represent those of their affiliated organizations, or those of the publisher, the editors and the reviewers. Any product that may be evaluated in this article, or claim that may be made by its manufacturer, is not guaranteed or endorsed by the publisher.
