# Supplementary material for: Efficacy and Safety of Pharmacological and Physical Therapies for Bell's Palsy: A Bayesian Network Meta-Analysis
Source: Front Neurol. 2022 Apr 18;13:868121. doi: 10.3389/fneur.2022.868121 (PMC9074786; doi:10.3389/fneur.2022.868121)
Supplement: Supplementary file 1 [file Data_Sheet_1.pdf]

Efficacy and safety of pharmacological and physical therapies for Bell's palsy: a Bayesian network meta-analysis

(Supplementary Files)

Contents

Tables

Table 1. Baseline characteristics for patients included in the study (Ster: Steroid; Anti: antiviral; Kaba: Kabat treatment; Elec: Electrical stimulation; Acup: Acupuncture; Faci: Facial exercise; NR: not reported; NA: not available; HBS: House-Brackmann rating scale; CR: criteria for recovery; AD: adverse events; S & C: synkinesis or crocodile tears) ……………………………………………………………………………………………………..…Pages 3-5

Table S2. Ranking of different treatments for facial recovery (Table S2A), adverse events (Table S2B) and sequelae (Table S2C). (A: steroids, B: antivirals, C: acupuncture, D: Kabat treatment, E: electrical stimulation, F: facial exercise, H: placebo)………………..…………………………………….………Pages 5-7

Table S3. Certainty of Evidence (GRADE) (Table S3A). Different treatments for facial recovery (Table S3B), adverse events (Table S3C) and sequelae (Table S3D). CI: Confidence interval; OR: Odds ratio. (Ster: Steroid; Anti: antiviral; Kaba: Kabat treatment; Elec: Electrical stimulation; Acup: Acupuncture; Faci: Facial exercise) ………………………………………………………………………………………………………...............................Pages 8-18

Table S4. Meta-regression assessing factors that contributed to the heterogeneity in effect size of studies that used different regimens to compared facial recovery after treatments (Table S4A). Detailed information collected for meta-regression analysis (Table S4B). (A: steroids, B: antivirals, C: acupuncture, D: Kabat treatment, E: electrical stimulation, F: facial exercise, H: placebo)…………………………………..…………………………………………Pages 19-20

Table S5. Summary of acupoints used for Bell's palsy in included studies. ……………………………………………………………..........Page 21

Figures

Figure S1. The flow chart. Study flow diagram illustrating the study selection process for this update…………………………………………………....Page 22

Figure S2. Forest plot of frequentist network meta-analysis using random-effects model for facial recovery (Figure S1A), adverse events (Figure S1B), and sequelae (Figure S1C). (Ster: Steroid; Anti: antiviral; Kaba: Kabat treatment; Elec: Electrical stimulation; Acup: Acupuncture; Faci: Facial exercise)………...Pages 23-24

Figure S3. Figure showing forest plot of inconsistency analysis according to facial recovery (Figure S2A), adverse events (Figure S2B), and sequelae (Figure

S2C). (A: steroids, B: antivirals, C: acupuncture, D: Kabat treatment, E: electrical stimulation, F: facial exercise, H: placebo) ……………………..Pages 25-26
Figure S4. Figure showing forest plot of heterogeneity analysis according to facial recovery (Figure S3A), adverse events (Figure S3B), and sequelae (Figure S3C). (A: steroids, B: antivirals, C: acupuncture, D: Kabat treatment, E: electrical stimulation, F: facial exercise, H: placebo) ……………...…..…Pages 27-29
Figure S5. Funnel plots and Egger's linear regression test (P-value) of facial recovery (Figure S4A), adverse events (Figure S4B), and sequelae (Figure S4C) with different treatments. ………..…Pages 30-32
Figure S6. Convergence of the three chains established by inspection of the history feature and the Brooks-Gelman-Rubin diagnostic: History for facial recovery (Figure S5A), adverse events (Figure S5B), and sequelae (Figure S5C); Brooks-Gelman-Rubin diagnostic for facial recovery (Figure S5D), adverse events (Figure S5E), and sequelae (Figure S5F). (A: steroids, B: antivirals, C: acupuncture, D: Kabat treatment, E: electrical stimulation, F: facial exercise, H: placebo) ………..Pages 33-38
Figure S7. Pooled estimates of the network meta-analysis for subgroup analysis. (A: Steroid, B:Antiviral, A1: prednisone, A2: prednisolone, A3: methylprednisolone, B1: acyclovir, B2: valaciclovir, B3: famciclovir, C: acupuncture, D: Kabat treatment, E: electrical stimulation, F: facial exercise, H: placebo) ……...Pages 39-40
Figure S8. Risk of bias graph for studies: review authors' judgements about each risk of bias item presented as percentages across all included studies. Page 41

Reference of included studies…………………………………………………………………………………………..…..Pages 42-44

Table S1

No.	Author, Year	Region	Sample	Sex, Male (%)	Age (Year, M±SD)	Time from onset of disease (Days)	HBS from onset of disease (Levels)	Treatment, No. of patients T1	T2	T3	T4	Follow-up (Months)	Main outcome HBS-CR	Secondary outcomes
1	Austin, 1993	America	76	39, (51)	37.1 ± 12.6	≤5	NR	Ster, 35	Placebo, 41	NA	NA	6	≤III	AD, S&C
2	Barbara, 2010	Italy	20	10, (50)	38.9	≤3	≥III	Ster+Anti+Kaba, 9	Ster+Anti, 11	NA	NA	2	≤III	NR
3	De Diego 1998	America	101	56, (55)	43.0	≤4	NR	Ster, 47	Anti, 54	NA	NA	12	≤II	S&C
4	Li, 2020	China	94	42, (45)	47.0 ± 12.0	≤7	≥III	Ster+Acup, 48	Ster, 46	NA	NA	1	≤II	NR
5	Engstrom, 2008	Sweden	829	488, (59)	40.7 ± 16.4	≤3	≥III	Ster+Anti, 206	Ster, 210	Anti, 207	Placebo, 206	12	≤I	AD, S&C
6	Hato, 2007	Japa	221	116, (52)	50.3	≤7	NR	Ster+Anti, 114	Ster, 107	NA	NA	6	≤I	NR
7	Khedr, 2016	Egypt	50	35, (70)	27.2 ± 4.5	≤3	≥IV	Ster+Anti, 25	Ster, 25	NA	NA	3	≤II	NR
8	Kim, 2016	Korea	60	NR	48.0 ± 18.0	≤7	≤IV	Ster+Anti+Elec, 30	Ster+Anti, 30	NA	NA	6	≤II	AD, S&C
9	Lee, 2013	Korea	206	101, (49)	47.7 ± 16.0	≤7	≥V	Ster+Anti, 99	Ster, 107	NA	NA	6	≤II	NR
10	Legalla, 2002	Italy	58	34, (55)	44.0 ± 19.6	≤3	≥III	Ster, 30	Placebo, 28	NA	NA	12	≤II	AD, S&C
11	Liang,	China	72	52,	55.0	≤14	≥III	Acup,	Placebo,	NA	NA	NR	≤II	NR

	2018			(48)	± 7.0			36	36					
12	Liu, 2010	China	131	64, (49)	37.5 ± 13.0	≤3	NR	Acup+Elec, 45	Ster+Anti+Acup, 42	Acup, 44	NA	3	≤III	NR
13	Monini, 2017	Italy	104	NR	54.5	≤7	≥IV	Ster+Kaba, 38	Ster, 66	NA	NA	NR	≤III	NR
14	Nicastri, 2013	Italy	87	44, (51)	49.0 ± 16.0	≤3	≥IV	Ster+Anti, 48	Ster+Anti+Faci, 39	NA	NA	6	≤II	S&C
15	Kawaguchi, 2007	Japan	150	46, (31)	54.5 ± 17.3	≤7	NR	Ster, 66	Ster+Anti, 84	NA	NA	6	≤I	NR
16	Adour, 1996	America	99	49, (49)	41.9 ± 14.1	≤3	NR	Ster+Anti, 53	Ster, 46	NA	NA	4	≤II	NR
17	Sullivan, 2007	Scotland	496	253, (51)	44.0 ± 16.4	≤3	NR	Ster+Anti, 124	Ster, 127	Anti, 123	Placebo, 122	9	≤I	AD
18	Tone, 2009	China	115	66, (55)	NR	≤12	≥III	Ster, 51	Acup, 27	Placebo, 37	NA	3	≤II	AD
19	Unuvar, 1999	Turkey	42	21, (50)	46.9 ± 4.7	≤3	≥IV	Ster, 21	Placebo, 21	NA	NA	12	≤II	NR
20	Xie, 2010	China	120	66, (55)	37.0 ± 21.0	≤7	NR	Acup, 60	Placebo, 60	NA	NA	4	≤III	NR
21	Yeo, 2008	Korea	91	21, (48)	42.7 ± 15.7	≤7	≥II	Ster+Anti, 44	Ster, 47	NA	NA	6	≤II	NR
22	Khajeh, 2015	Iran	43	23, (53)	8.5 ± 5.0	≤7	NR	Ster+Anti, 20	Ster, 23	NA	NA	6	≤I	NR
23	Minnerop, 2008	German y	117	60, (51)	40.6 ± 20.5	≤5	≥III	Ster+Anti, 50	Ster, 67	NA	NA	12	≤I	AD
24	Qu,	China	90	45,	42.5	≤7	NR	Acup+Faci,	Acup,	Faci,	NA	2	≤III	NR

	2005			(50)				30	30	30				
25	Xu, 2020	China	64	38, (59)	39.0 ± 19.0	≤7	NR	Ster, 32	Acup, 32	NA	NA	1	≤III	NR
26	Ferreira, 2016	Portugal	73	35, (48)	42.4	≤3	NR	Ster+Faci, 42	Faci, 31	NA	NA	6	≤I	NR

Table S2A

	Rank 1	Rank 2	Rank 3	Rank 4	Rank 5	Rank 6	Rank 7	Rank 8	Rank 9	Rank 10	Rank 11	Rank 12	Rank 13	Rank 14	SUCRA
A	0	0	0	1.67E-05	0.000242	0.001433	0.005808	0.018808	0.054808	0.141633	0.273917	0.265725	0.227233	0.009733	0.256
A+B	0	0.000608	0.004225	0.019383	0.052767	0.106583	0.157467	0.192583	0.193475	0.1541	0.0857	0.032925	0.000175	0	0.481
A+B+C	0.010017	0.042567	0.082283	0.11065	0.125317	0.120325	0.104217	0.093767	0.088092	0.077242	0.06085	0.0405	0.02545	0.010942	0.576
A+B+D	0.304908	0.177542	0.123542	0.091942	0.068358	0.052542	0.039383	0.032758	0.029333	0.025442	0.020358	0.014958	0.010142	0.004717	0.802
A+B+E	0.234525	0.169858	0.133792	0.105592	0.081525	0.059975	0.049617	0.0406	0.035283	0.030617	0.022567	0.016783	0.010625	0.004275	0.773
A+B+F	0.059542	0.105475	0.13045	0.134025	0.124092	0.100658	0.079983	0.066742	0.060575	0.051475	0.0384	0.02645	0.01375	0.004992	0.670
A+C	0.034758	0.065625	0.090133	0.106658	0.110283	0.10315	0.089617	0.082	0.081283	0.079475	0.062392	0.04715	0.026483	0.011733	0.590
A+D	0.050825	0.076617	0.092125	0.101367	0.100442	0.091692	0.084183	0.076642	0.076492	0.077842	0.064075	0.050883	0.030542	0.014275	0.593
A+F	0.00345	0.006883	0.010758	0.015517	0.01975	0.025275	0.029275	0.034817	0.04225	0.05535	0.078267	0.107267	0.13565	0.093	0.205
B	0	0	0	0.000025	0.000008	0.000175	0.00035	0.00105	0.003075	0.008675	0.0303	0.102825	0.186783	0.3246	0.086
C	0.000102	0.001358	0.009325	0.034283	0.081075	0.139842	0.188592	0.200908	0.173083	0.1113	0.044375	0.013208	0.002283	0.000233	0.522
**C+E**	0.219492	0.236525	0.188825	0.131775	0.086067	0.0525	0.032158	0.020275	0.01295	0.008817	0.005567	0.002792	0.001425	0.000492	0.839
C+F	0.08035	0.109183	0.1177	0.119958	0.109458	0.09325	0.077525	0.067583	0.064192	0.060783	0.045733	0.024467	0.017942	0.0079	0.664
F	0.002033	0.007758	0.016842	0.028808	0.040608	0.0526	0.061792	0.071208	0.084042	0.112208	0.1416	0.150533	0.085642	0.119292	0.350
H	0	0	0	0	0.000008	0	0.000033	0.000258	0.001067	0.005042	0.0259	0.103533	0.225875	0.393817	0.092

Table S2B

	Rank 1	Rank 2	Rank 3	Rank 4	Rank 5	Rank 6	SUCRA
A	0.001017	0.020117	0.181025	0.493383	0.233192	0.071267	0.370
A+B	0.0019	0.104908	0.679575	0.15215	0.047425	0.014042	0.564
A+B+E	0.705733	0.269408	0.010683	0.005858	0.003733	0.004583	0.931
**B**	0.000558	0.007017	0.043942	0.149642	0.297633	0.501208	0.152
C	0.290625	0.595467	0.045425	0.02205	0.016225	0.030208	0.806
H	0.000167	0.003083	0.03935	0.176917	0.401792	0.378692	0.177

Table S2C

	Rank 1	Rank 2	Rank 3	Rank 4	Rank 5	Rank 6	SUCRA
A	0.023075	0.094592	0.362867	0.4353	0.071108	0.013058	0.505
A+B	0.001283	0.007875	0.021083	0.116167	0.742892	0.1107	0.215
**A+B+E**	0.014867	0.0132	0.015358	0.033008	0.073275	0.850292	0.063
A+B+F	0.263858	0.13055	0.195067	0.303433	0.087142	0.01995	0.624
B	0.32325	0.379533	0.217342	0.061633	0.014492	0.00375	0.785
H	0.373667	0.37425	0.188283	0.050458	0.011092	0.00225	0.808

Table S3A

Direct Comparison	study limitations	inconsistency	indirectness	imprecision	publication bias	GRADE
Anti vs Ster	not serious	not serious	serious	not serious	not serious	moderate
Anti vs Ster+Anti	not serious	not serious	serious	not serious	not serious	moderate
Acup vs Ster	not serious	serious	not serious	serious	not serious	low
Acup vs Ster+Anti+Acup	serious	not serious	not serious	serious	not serious	low
Faci vs Acup	serious	not serious	not serious	serious	not serious	low
Faci vs Ster+Faci	not serious	not serious	not serious	serious	not serious	moderate
Placebo vs Anti	not serious	not serious	serious	not serious	not serious	moderate
Placebo vs Acup	not serious	not serious	serious	serious	not serious	low
Placebo vs Ster	serious	not serious	serious	not serious	not serious	low
Placebo vs Ster+Anti	not serious	not serious	serious	not serious	not serious	moderate
Ster+Anti vs Ster	not serious	serious	not serious	not serious	not serious	moderate
Ster+Acup vs Ster	serious	not serious	not serious	serious	not serious	low
Ster+Faci vs Ster	serious	not serious	not serious	serious	not serious	low
Acup+Elec vs Acup	serious	not serious	not serious	serious	not serious	low
Acup+Elec vs Ster+Anti+Acup	serious	not serious	not serious	serious	not serious	low
Acup+Faci vs Acup	serious	not serious	not serious	serious	not serious	low
Ster+Anti+Kaba vs Ster+Anti	serious	not serious	not serious	serious	not serious	low
Ster+Anti+Elec vs Ster+Anti	not serious	not serious	not serious	serious	not serious	moderate
Ster+Anti+Faci vs Ster+Anti	not serious	not serious	not serious	serious	not serious	moderate

Table S3B

Comparison	No. of comparisons	Direct evidence			Indirect evidence			Network meta-analysis			P Value for test of inconsistency
		OR (95% CI)	I2 (%)	Certainty of evidence	OR (95% CI)	I2 (%)	Certainty of evidence	OR (95% CI)	I2 (%)	Certainty of evidence	
Ster vs Faci	0	NA	NA	—	0.79 (0.12, 5.34)	NA	Low	0.79 (0.12, 5.34)	NA	Low	NA
Ster vs Ster+Faci	0	NA	NA	—	0.68 (0.06, 7.49)	NA	Low	0.68 (0.062, 7.49)	NA	Low	NA
Ster vs Acup+Elec	0	NA	NA	—	0.13 (0.02, 0.76)	NA	Low	0.13 (0.02, 0.76)	NA	Low	NA
Ster vs Acup+Faci	0	NA	NA	—	0.27 (0.03, 2.04)	NA	Low	0.27 (0.03, 2.04)	NA	Low	NA
Ster vs Ster+Anti+Acup	0	NA	NA	—	0.5 (0.02, 3.34)	NA	Low	0.25 (0.018, 3.34)	NA	Low	NA
Ster vs Ster Anti Kaba	0	NA	NA	—	0.13 (0.01, 1.22)	NA	Low	0.13 (0.01, 1.22)	NA	Low	NA
Ster vs Ster+Anti+Elec	0	NA	NA	—	0.16 (0.01, 1.22)	NA	Moderate	0.6 (0.01, 1.22)	NA	Moderate	NA
Anti vs Ster	3	0.45 (0.19, 0.86)	72.3	Moderate	NA	NA	—	0.51 (0.24, 0.98)	70.3	Moderate	NA
Anti vs Acup	0	NA	NA	—	0.23 (0.07, 0.64)	NA	Low	0.23 (0.07, 0.64)	NA	Low	NA
Anti vs Faci	0	NA	NA	—	0.41 (0.05, 2.84)	NA	Low	0.41 (0.05, 2.84)	NA	Low	NA
Anti vs Ster+Anti	2	0.49	55.3	Moderate	0.01	NA	Moderate	0.26	96	Moderate	NA

		(0.21, 1.1)			(0.01,0.01)			(0.11, 0.51)			
Anti vs Ster+Faci	0	NA	NA	—	0.74 (0.06, 8.52)	NA	Low	0.74 (0.06, 8.52)	NA	Low	NA
Acup vs Ster	2	2.7 (0.8,10.0)	7.7	Low	1.8 (0.45, 7.2)	NA	Low	2.3 (0.90, 5.8)	0	Low	0.769
Acup vs Ster+Anti	0	NA	NA	—	1.12 (0.39, 3.04)	NA	Low	1.12 (0.39, 3.04)	NA	Low	NA
Acup vs Ster+Faci	0	NA	NA	—	3.31 (0.37, 30.13)	NA	Low	3.31 (0.37, 30.12)	NA	Low	NA
Acup vs Ster+Anti+Acup	1	0.79 (0.23, 2.9)	NA	Low	NA	NA	—	0.82 (0.20, 3.4)	NA	Low	NA
Faci vs Acup	1	0.55 (0.11, 2.7)	NA	Low	NA	NA	—	0.57 (0.11, 2.9)	NA	Low	NA
Faci vs Placebo	0	NA	NA	—	2.37 (0.37, 15.66)	NA	Low	2.37 (0.37, 15.66)	NA	Low	NA
Faci vs Ster+Faci	1	1.8 (0.53, 6.5)	NA	Moderate	NA	NA	—	1.9 (0.43, 8.1)	NA	Moderate	NA
Faci vs Acup+Faci	1	0.34 (0.06, 2.2)	NA	Low	NA	NA	—	0.34 (0.056, 1.8)	NA	Low	NA
Placebo vs Anti	2	1.0 (0.49, 2.1)	67.3	Moderate	NA	NA	—	1.0 (0.49, 2.3)	68.6	Moderate	0.474
Placebo vs Acup	3	0.27 (0.11, 0.64)	0	Low	0.06 (0.002, 0.72)	NA	Low	0.24 (0.09, 0.54)	NA	Low	0.264
Placebo vs Ster	6	0.44 (0.23, 0.79)	22.1	Low	3.7 (0.51, 27.0)	NA	Moderate	0.54 (0.28, 0.93)	34.2	Moderate	0.275
Placebo	2	0.46	0	Moderate	0.077	NA	Moderate	0.27	75.4	Moderate	0.074

vs Ster+Anti		(0.20,1.1)			(0.022, 0.27)			(0.12, 0.49)			
Ster+Anti vs Ster	10	2.3 (1.5, 3.6)	5.5	Moderate	NA	NA	—	2.0 (1.3, 3.3)	6.7	Moderate	0.033
Ster+Anti vs Faci	0	NA	NA	—	1.6 (0.24, 11.4)	NA	Low	1.6 (0.24, 11.4)	NA	Low	NA
Ster+Anti vs Ster+Faci	0	NA	NA	—	2.94 (0.27, 34.64)	NA	Low	2.94 (0.27, 34.64)	NA	Low	NA
Ster+Acup vs Ster	1	2.8 (0.71, 12.0)	NA	Low	NA	NA	—	2.9 (0.59, 15.0)	NA	Low	NA
Ster+Acup vs Anti	0	NA	NA	—	5.57 (1.03, 34.8)	NA	Low	5.57 (1.03, 34.8)	NA	Low	NA
Ster+Acup vs Acup	0	NA	NA	—	1.27 (0.2, 8.38)	NA	Low	1.27 (0.2, 8.38)	NA	Low	NA
Ster+Acup vs Faci	0	NA	NA	—	2.29 (0.19, 27.37)	NA	Low	2.29 (0.19, 27.37)	NA	Low	NA
Ster+Acup vs Placebo	0	NA	NA	—	5.34 (1.02, 31.87)	NA	Low	5.34 (1.02, 31.87)	NA	Low	NA
Ster+Acup vs Ster+Anti	0	NA	NA	—	1.42 (0.26, 7.69)	NA	Low	1.42 (0.26, 7.69)	NA	Low	NA
Ster+Acup vs Ster+Faci	0	NA	NA	—	4.23 (0.23, 76.9)	NA	Low	4.23 (0.23, 76.9)	NA	Low	NA
Ster+Acup vs Ster+Anti+Acup	0	NA	NA	—	1.03 (0.10, 10.93)	NA	Low	1.03 (0.1, 10.93)	NA	Low	NA
Ster+Faci vs Ster	1	2.8 (0.63, 17.0)	NA	Low	NA	NA	—	2.8, (0.53,19.0)	NA	Low	NA
Ster+Faci vs Anti	0	NA	NA	—	5.64	NA	Low	5.6	NA	Low	NA

					(0.94, 45.94)			(0.94, 45.94)			
Ster+Faci vs Acup	0	NA	NA	—	1.28 (0.18, 10.94)	NA	Low	1.28 (0.18, 10.94)	NA	Low	NA
Ster+Faci vs Faci	0	NA	NA	—	2.32 (0.18, 34.37)	NA	Low	2.32 (0.18, 34.37)	NA	Low	NA
Ster+Faci vs Placebo	0	NA	NA	—	5.39 (0.92, 42.08)	NA	Low	5.39 (0.92, 42.08)	NA	Low	NA
Ster+Faci vs Ster+Anti	0	NA	NA	—	1.43 (0.24, 10.21)	NA	Low	1.43 (0.24, 10.21)	NA	Low	NA
Ster+Faci vs Ster+Acup	0	NA	NA	—	1.01 (0.09, 12.2)	NA	Low	1.01 (0.09, 12.2)	NA	Low	NA
Ster+Faci vs Ster+Faci	0	NA	NA	—	4.25 (0.22, 91.6)	NA	Low	4.25 (0.22, 91.6)	NA	Low	NA
Ster+Faci vs Ster+Anti+Acup	0	NA	NA	—	1.05 (0.09, 13.68)	NA	Low	1.05 (0.09, 13.68)	NA	Low	NA
Acup+Elec vs Anti	0	NA	NA	—	14.58 (2.48, 94.18)	NA	Low	14.58 (2.48, 94.18)	NA	Low	NA
Acup+Elec vs Acup	1	3.3 (0.82, 13.0)	NA	Low	NA	NA	—	3.3 (0.79, 14.0)	NA	Low	NA
Acup+Elec vs Faci	0	NA	NA	—	5.921 (0.677, 53.66)	NA	Low	5.921 (0.677, 53.66)	NA	Low	NA
Acup+Elec vs Placebo	0	NA	NA	—	13.97 (2.669, 79.171)	NA	Low	13.97 (2.669, 79.171)	NA	Low	NA
Acup+Elec vs Ster+Anti	0	NA	NA	—	3.723 (0.612, 21.074)	NA	Low	3.723 (0.612, 21.074)	NA	Low	NA
Acup+Elec	0	NA	NA	—	2.612 (0.241,	NA	Low	2.612 (0.241,	NA	Low	NA

vs Ster+Acup					27.577)			27.577)			
Acup+Elec vs Ster+Kaba	0	NA	NA	—	2.55 (0.19, 29.21)	NA	Low	2.55 (0.19, 29.21)	NA	Low	NA
Acup+Elec vs Ster+Faci	0	NA	NA	—	10.95 (0.81, 152.6)	NA	Low	10.95 (0.81, 152.6)	NA	Low	NA
Acup+Elec vs Acup+Faci	0	NA	NA	—	2.01 (0.19, 20.2)	NA	Low	2.01 (0.19, 20.2)	NA	Low	NA
Acup+Elec vs Ster+Anti+Acup	1	2.7 (0.65, 11.0)	NA	Low	NA	NA	—	2.7 (0.65, 12)	NA	Low	NA
Acup+Elec vs Ster+Anti+Elec	0	NA	NA	—	1.16 (0.06, 16.87)	NA	Low	1.16 (0.06, 16.87)	NA	Low	NA
Acup+Elec vs Ster+Anti+Faci	0	NA	NA	—	1.96 (0.18, 19.88)	NA	Low	1.96 (0.178, 19.88)	NA	Low	NA
Acup+Faci vs Anti	0	NA	NA	—	7.29 (0.92, 64.49)	NA	Low	7.29 (0.92, 64.49)	NA	Low	NA
Acup+Faci vs Acup	1	1.7 (0.21, 13.0)	NA	Low	NA	NA	—	1.7 (0.29, 10.0)	NA	Low	NA
Acup+Faci vs Placebo	0	NA	NA	—	6.97 (0.97, 55.67)	NA	Low	6.97 (0.97, 55.67)	NA	Low	NA
Acup+Faci vs Ster+Anti	0	NA	NA	—	1.84 (0.23, 15)	NA	Low	1.84 (0.23, 15)	NA	Low	NA
Acup+Faci vs Ster+Acup	0	NA	NA	—	1.3 (0.09, 17.89)	NA	Low	1.3 (0.09, 17.89)	NA	Low	NA
Acup+Faci vs Ster+Kaba	0	NA	NA	—	1.26 (0.08, 18.99)	NA	Low	1.26 (0.08, 18.99)	NA	Low	NA
Acup+Faci	0	NA	NA	—	5.43	NA	Low	5.43	NA	Low	NA

vs Ster+Faci					(0.57, 55.42)			(0.57, 55.42)			
Acup+Faci vs Ster+Anti+Acup	0	NA	NA	—	1.35 (0.14, 13.73)	NA	Low	1.35 (0.14, 13.73)	NA	Low	NA
Ster+Anti+Kaba vs Anti	0	NA	NA	—	15.44 (1.56, 189.62)	NA	Low	15.44 (1.56, 189.62)	NA	Low	NA
Ster+Anti+Kaba vs Acup	0	NA	NA	—	3.47 (0.31, 45.95)	NA	Low	3.47 (0.31, 45.95)	NA	Low	NA
Ster+Anti+Kaba vs Faci	0	NA	NA	—	6.26 (0.34, 136.32)	NA	Low	6.26 (0.34, 136.32)	NA	Low	NA
Ster+Anti+Kaba vs Placebo	0	NA	NA	—	14.78 (1.49, 178.79)	NA	Low	14.78 (1.49, 178.79)	NA	Low	NA
Ster+Anti+Kaba vs Ster+Anti	1	3.7 (0.47, 35.0)	NA	Low	NA	NA	—	3.8 (0.43, 40.0)	NA	Low	NA
Ster+Anti+Kaba vs Ster+Acup	0	NA	NA	—	2.75 (0.17, 51.21)	NA	Low	2.75 (0.17, 51.21)	NA	Low	NA
Ster+Anti+Kaba vs Ster+Kaba	0	NA	NA	—	2.69 (0.14, 51.72)	NA	Low	2.69 (0.14, 51.72)	NA	Low	NA
Ster+Anti+Kaba vs Ster+Faci	0	NA	NA	—	11.64 (0.44, 348.61)	NA	Low	11.64 (0.44, 348.61)	NA	Low	NA
Ster+Anti+Kaba vs Acup+Faci	0	NA	NA	—	2.1 (0.1, 49.53)	NA	Low	2.1 (0.1, 49.53)	NA	Low	NA
Ster+Anti+Kaba vs Ster+Anti+Acup	0	NA	NA	—	2.861 (0.17, 53.44)	NA	Low	2.861 (0.17, 53.44)	NA	Low	NA
Ster+Anti+Kaba vs Ster+Anti+Elec	0	NA	NA	—	1.195 (0.05, 27.78)	NA	Low	1.195 (0.5, 27.78)	NA	Low	NA
Ster+Anti+Kaba	0	NA	NA	—	2.056	NA	Low	2.056 (0.133,	NA	Low	NA

vs Ster+Anti+Faci					(0.13, 33.8)			33.805)			
Ster+Anti+Elec vs Anti	0	NA	NA	—	12.514 (1.52, 156.76)	NA	Moderate	12.51 (1.52, 156.76)	NA	Moderate	NA
Ster+Anti+Elec vs Acup	0	NA	NA	—	2.829 (0.3, 37.16)	NA	Low	2.829 (0.3, 37.16)	NA	Low	NA
Ster+Anti+Elec vs Faci	0	NA	NA	—	5.154 (0.32, 107.24)	NA	Low	5.154 (0.32, 107.24)	NA	Low	NA
Ster+Anti+Elec vs Placebo	0	NA	NA	—	11.979 (1.49,145.08)	NA	Moderate	11.979 (1.49,145.1)	NA	Moderate	NA
Ster+Anti+Elec vs Ster+Anti	1	3.3 (0.50, 22.0)	NA	Moderate	NA	NA	—	3.2 (0.41, 36.)	NA	Moderate	NA
Ster+Anti+Elec vs Ster+Acup	0	NA	NA	—	2.256 (0.16, 40.36)	NA	Low	2.256 (0.16, 40.36)	NA	Low	NA
Ster+Anti+Elec vs Ster+Kaba	0	NA	NA	—	2.219 (0.128, 42.61)	NA	Low	2.219 (0.13, 42.61)	NA	Low	NA
Ster+Anti+Elec vs Ster+Faci	0	NA	NA	—	9.6 (0.413, 271)	NA	Low	9.6 (0.413, 271.1)	NA	Low	NA
Ster+Anti+Elec vs Acup+Faci	0	NA	NA	—	1.696 (0.127, 27.812)	NA	Low	1.696 (0.127, 27.81)	NA	Low	NA
Ster+Anti+Elec vs Ster+Anti+Acup	0	NA	NA	—	2.345 (0.167, 43.93)	NA	Low	2.345 (0.167, 43.93)	NA	Low	NA
Ster+Anti+Elec vs Ster+Anti+Faci	0	NA	NA	—	1.748 (0.095, 39.15)	NA	Moderate	1.748 (0.095, 39.16)	NA	Moderate	NA
Ster+Anti+Faci vs Ster+Anti	1	1.9 (0.50, 7.6)	NA	Moderate	NA	NA	—	1.9 (0.41, 9.0)	NA	Moderate	NA

Table S3C

Comparison	No. of comparisons	Direct evidence			Indirect evidence			Network meta-analysis			P Value for test of inconsistency
		OR (95% CI)	I2 (%)	Certainty of evidence	OR (95% CI)	I2 (%)	Certainty of evidence	OR (95% CI)	I2 (%)	Certainty of evidence	
Anti vs Ster	2	0.86 (0.33, 2.2)	0	Moderate	NA	NA	—	0.74 (0.3, 1.6)	0	Moderate	NA
Anti vs Ster+Anti	2	0.57 (0.24, 1.3)	0	Moderate	NA	NA	—	0.56 (0.23, 1.3)	0	Moderate	NA
Acup vs Ster	1	9.2 (0.42, 2.0e+02)	NA	Low	NA	NA	—	5.5 (0.66, 55.0)	NA	Low	NA
Acup vs Anti	0	NA	NA	—	7.44 (0.712, 92.729)	NA	Low	7.44 (0.712, 92.729)	NA	Low	NA
Acup vs Ster+Anti	0	NA	NA	—	4.102 (0.366, 50.265)	NA	Low	4.102 (0.366, 50.265)	NA	Low	NA
Placebo vs Ster	5	0.67 (0.29, 1.3)	15.3	Low	NA	NA	—	0.78 (0.34, 1.5)	11.5	Low	NA
Placebo vs Anti	2	1.2 (0.48, 3.3)	0	Moderate	NA	NA	—	1.1 (0.42, 2.3)	0	Moderate	NA
Placebo vs Acup	1	0.13 (0.005, 3.6)	NA	Low	NA	NA	—	0.14 (0.013, 1.2)	NA	Low	NA
Placebo vs Ster+Anti	2	0.7 (0.31, 1.6)	0	Moderate	6.9e-09 (1.5e-12, 3.2e-05)	NA	Moderate	0.59 (0.24, 1.2)	89.2	Moderate	0.164
Ster+Anti vs Ster	3	1.5 (0.73, 3.4)	0	Moderate	NA	NA	—	1.3 (0.6, 2.8)	0	Moderate	NA
Ster+Anti+Elec vs	0	NA	NA	—	13.883 (1.279,	NA	Low	13.883 (1.279,	NA	Low	NA

Ster					417.666)			417.666)			
Ster+Anti+Elec vs Anti	0	NA	NA	—	18.659 (1.723, 578.284)	NA	Moderate	18.659 (1.723, 578.284)	NA	Moderate	NA
Ster+Anti+Elec vs Acup	0	NA	NA	—	2.594 (0.084, 142.24)	NA	Low	2.594 (0.084, 142.24)	NA	Low	NA
Ster+Anti+Elec vs Placebo	0	NA	NA	—	17.93 (1.695, 569.781)	NA	Moderate	17.93 (1.695, 569.781)	NA	Moderate	NA
Ster+Anti+Elec vs Ster+Anti	1	9.2 (1.1, 2.3e+02)	NA	Moderate	NA	NA	—	11.0 (1.0, 3.4e+02)	NA	Moderate	NA

Table S3D

Comparison	No. of comparisons	Direct evidence			Indirect evidence			Network meta-analysis			P Value for test of inconsistency
		OR (95% CI)	I2 (%)	Certainty of evidence	OR (95% CI)	I2 (%)	Certainty of evidence	OR (95% CI)	I2 (%)	Certainty of evidence	
Anti vs Ster	2	0.86 (0.33, 2.2)	0	Moderate	NA	NA	—	0.74 (0.3, 1.6)	0	Moderate	NA
Anti vs Ster+Anti	2	0.57 (0.24, 1.3)	0	Moderate	NA	NA	—	0.56 (0.23, 1.3)	0	Moderate	NA
Acup vs Ster	1	9.2 (0.42, 2.0e+02)	NA	Low	NA	NA	—	5.5 (0.66, 55.0)	NA	Low	NA
Acup vs Anti	0	NA	NA	—	7.44 (0.712, 92.729)	NA	Low	7.44 (0.712, 92.729)	NA	Low	NA
Acup vs Ster+Anti	0	NA	NA	—	4.102 (0.366, 50.265)	NA	Low	4.102 (0.366, 50.265)	NA	Low	NA
Placebo vs Ster	5	0.67 (0.29,	15.3	Low	NA	NA	—	0.78 (0.34, 1.5)	11.5	Low	NA

		1.3)									
Placebo vs Anti	2	1.2 (0.48, 3.3)	0	Moderate	NA	NA	—	1.1 (0.42, 2.3)	0	Moderate	NA
Placebo vs Acup	1	0.13 (0.005, 3.6)	NA	Low	NA	NA	—	0.14 (0.013, 1.2)	NA	Low	NA
Placebo vs Ster+Anti	2	0.7 (0.31, 1.6)	0	Moderate	6.9e-09 (1.5e-12, 3.2e-05)	NA	Moderate	0.59 (0.24, 1.2)	89.2	Moderate	0.164
Ster+Anti vs Ster	3	1.5 (0.73, 3.4)	0	Moderate	NA	NA	—	1.3 (0.6, 2.8)	0	Moderate	NA
Ster+Anti+Elec vs Ster	0	NA	NA	—	13.883 (1.279, 417.666)	NA	Low	13.883 (1.279, 417.666)	NA	Low	NA
Ster+Anti+Elec vs Anti	0	NA	NA	—	18.659 (1.723, 578.284)	NA	Moderate	18.659 (1.723, 578.284)	NA	Moderate	NA
Ster+Anti+Elec vs Acup	0	NA	NA	—	2.594 (0.084, 142.24)	NA	Low	2.594 (0.084, 142.24)	NA	Low	NA
Ster+Anti+Elec vs Placebo	0	NA	NA	—	17.93 (1.695, 569.781)	NA	Moderate	17.93 (1.695, 569.781)	NA	Moderate	NA
Ster+Anti+Elec vs Ster+Anti	1	9.2 (1.1, 2.3e+02)	NA	Moderate	NA	NA	—	11.0 (1.0, 3.4e+02)	NA	Moderate	NA

Table S4A

	Estimate	95% confidence interval	Se	z-val	p-val
Follow-up	0.006	(-0.053 – 0.065)	0.030	0.200	0.841
Age	-0.016	(-0.038 – 0.006)	0.011	-1.384	0.167
Sex	-0.009	(-0.033 – 0.015)	0.012	-0.751	0.452
Publication year	0.013	(-0.013 – 0.039)	0.013	0.991	0.322
Country	0.011	(-0.354 – 0.375)	0.186	0.057	0.955

k = 26; I^2 (residual heterogeneity / unaccounted variability): 0.00%; H^2 (unaccounted variability / sampling variability): 0.57

TableS4B

Author, year	Mean HBS level (baseline)	Follow up (months)	Country	Mean age (years)	Sex (male rate, %)	Treatment1	n1	event1	Treatment2	n2	event2	Treatment3	n3	event3	Treatment4	n4	event4
Austin, 1993	4.2	6	America	37.1	51	A	35	35	H	41	34						
Barbara, 2010	4	2	Italy	38.9	50	A+B+D	9	5	A+B	11							
De Diego 1998	NR	12	America	43	55	A	47	44	B	54	42						
Li, 2020	4	1	China	47	45	A+C	48	43	A	46	35						
Engstrom, 2008	4	12	Sweden	40.7	59	A+B	206	152	A+H	210	148	B+H	207	135	H	206	118
Hato, 2007	NR	6	Japa	50.3	52	A+B	114	110	A+H	107	96						
Khedr, 2016	4.9	3	Egypt	27.2	70	A+B	25	23	A	25	17						
Kim, 2016	3.45	6	Korea	48	NR	A+B+E	30	28	A+B	30	25						
Lee, 2013	5	6	Korea	47.7	49	A+B	99	82	A	107	71						
Legalla, 2002	3.73	12	Italy	44	55	A+H	30	25	H	28	21						
Liang, 2018	4.79	NR	China	55	48	A+H	36	31	H	36	22	C	36	28			
Liu, 2010	NR	3	China	37.5	49	C+E	45	35	A+B+C	42	24	C	44	23			
Monini, 2017	4.14	NR	Italy	54.5	NR	A+D	38	35	A	66	54						

Nicastri, 2013	5.36	6	Italy	49	51	A+B	48	36	A+B+F	39	33						
Kawaguchi, 2007	NR	6	Japan	54.5	31	A	66	55	A+B	84	78						
Adour, 1996	NR	4	America	41.9	49	A+B	53	49	A	46	35						
Sullivan, 2007	NR	9	Scotland	44	51	A+B	124	115	A	127	122	B	123	96	H	122	104
Tone, 2009	3.87	3	China	NR	55	A	51	34	C	27	21	H	37	22			
Unuvar, 1999	4.5	12	Turkey	46.9	50	A	21	21	H	21	20						
Xie, 2010	NR	4	China	37	55	C	60	57	H	60	49						
Yeo, 2008	3.71	6	Korea	42.7	48	A+B	44	41	A	47	40						
Khajeh, 2015	NR	6	Iran	8.5	53	A+B	20	18	A	23	15						
Minnerop, 2008	3.99	12	Germany	40.6	51	A+B	50	41	A	67	47						
Qu, 2005	NR	2	China	42.5	50	C+F	30	26	C	30	24	F	30	21			
Xu, 2020	3.75	1	China	39	59	A+H	32	27	C	32	31						
Ferreira, 2016	4.33	6	Portugal	42.4	48	A+F	42	27	F	31	20						

Table S5

No.	Author, Year	Region	Acupoints
4	Li, 2020	China	Ipsilateral: Quanliao (SI18), Dicang (ST4), Jiache (ST6), Yangbai (GB14), Sibai (ST2), Yifeng (TE17), Cuanzhu (BL2), Chengjiang (CV24), Yingxiang (LI20). Opposite: Hegu (LI4).
12	Liu, 2010	China	Ipsilateral: Quanliao (SI18), Dicang (ST4), Jiache (ST6), Yangbai (GB14), Yifeng (TE17), Taiyang (EX-HN7), Cuanzhu (BL2), Chengjiang (CV24), Yingxiang (LI20), Xiaguan (ST7), Zusanli (ST36). Opposite: Hegu (LI4).
18	Tone, 2009	China	Ipsilateral: Quanliao (SI18), Dicang (ST4), Jiache (ST6), Yangbai (GB14), Sibai (ST2), Yifeng (TE17), Taiyang (EX-HN7), Hegu (LI4).
20	Xie, 2010	China	Ipsilateral: Dicang (ST4), Jiache (ST6), Yangbai (GB14), Yifeng (TE17),Taiyang (EX-HN7),Cuanzhu (BL2), Wangu (GB12). Opposite: Hegu (LI4).
24	Qu, 2005	China	Ipsilateral: Dicang (ST4), Jiache (ST6), Yangbai (GB14), Sibai (ST2), Yingxiang (LI20), Yuyao (EX-HN4), Hegu (LI4). Opposite: Hegu (LI4).
25	Xu, 2020	China	Ipsilateral: Dicang (ST4), Jiache (ST6), Yangbai (GB14), Yifeng (TE17), Cuanzhu (BL2), Yingxiang (LI20), Yuyao (EX-HN4). Opposite: Hegu (LI4).

Figure S1.

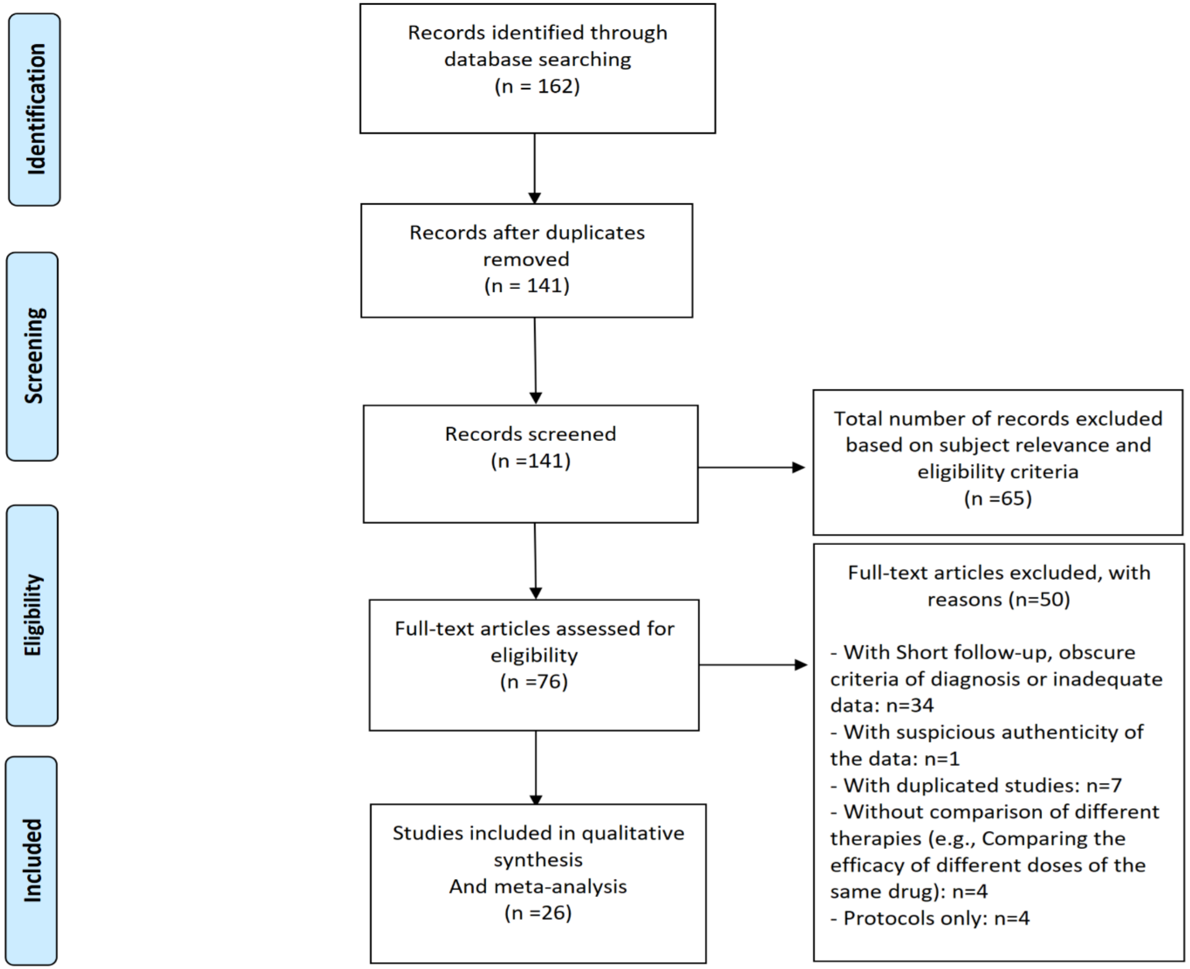

Identification
Screening
Eligibility
Included
Records identified through database searching (n = 162)
Records after duplicates removed (n = 141)
Records screened (n =141)
Total number of records excluded based on subject relevance and eligibility criteria (n =65)
Full-text articles assessed for eligibility (n =76)
Full-text articles excluded, with reasons (n=50)
- With Short follow-up, obscure criteria of diagnosis or inadequate data: n=34
- With suspicious authenticity of the data: n=1
- With duplicated studies: n=7
- Without comparison of different therapies (e.g., Comparing the efficacy of different doses of the same drug): n=4
- Protocols only: n=4
Studies included in qualitative synthesis And meta-analysis (n =26)

Figure S2.

Figure S2A

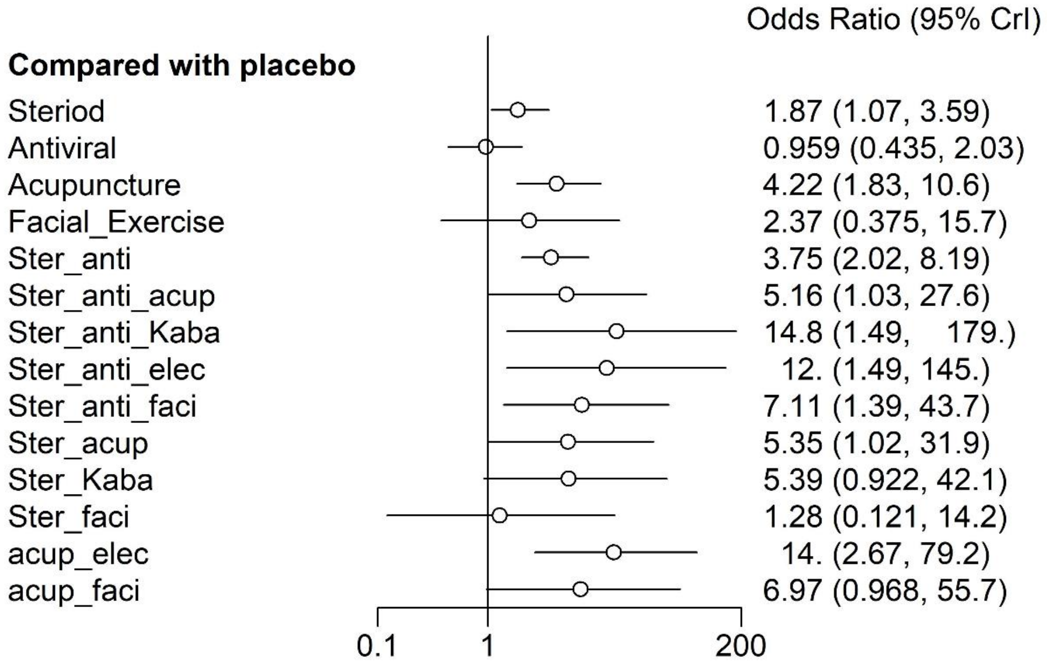

Figure S2B

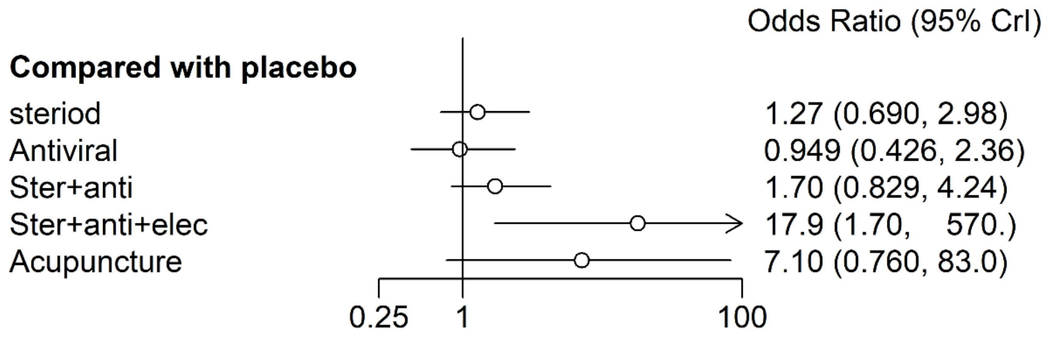

Figure S2C

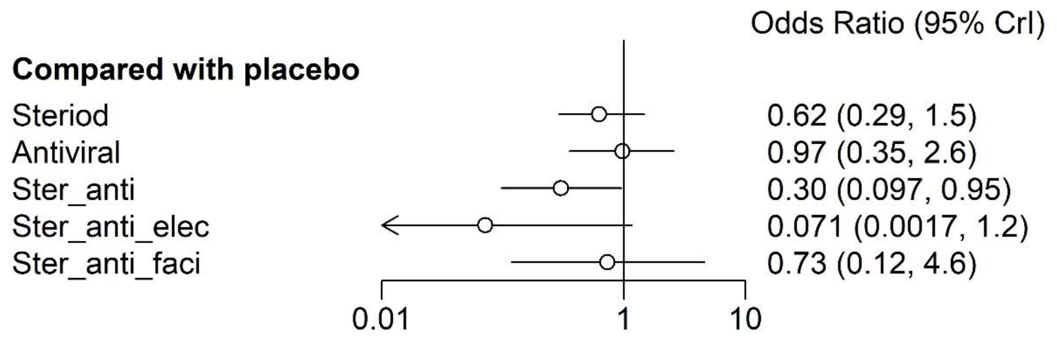

FigureS3

Figure S3A

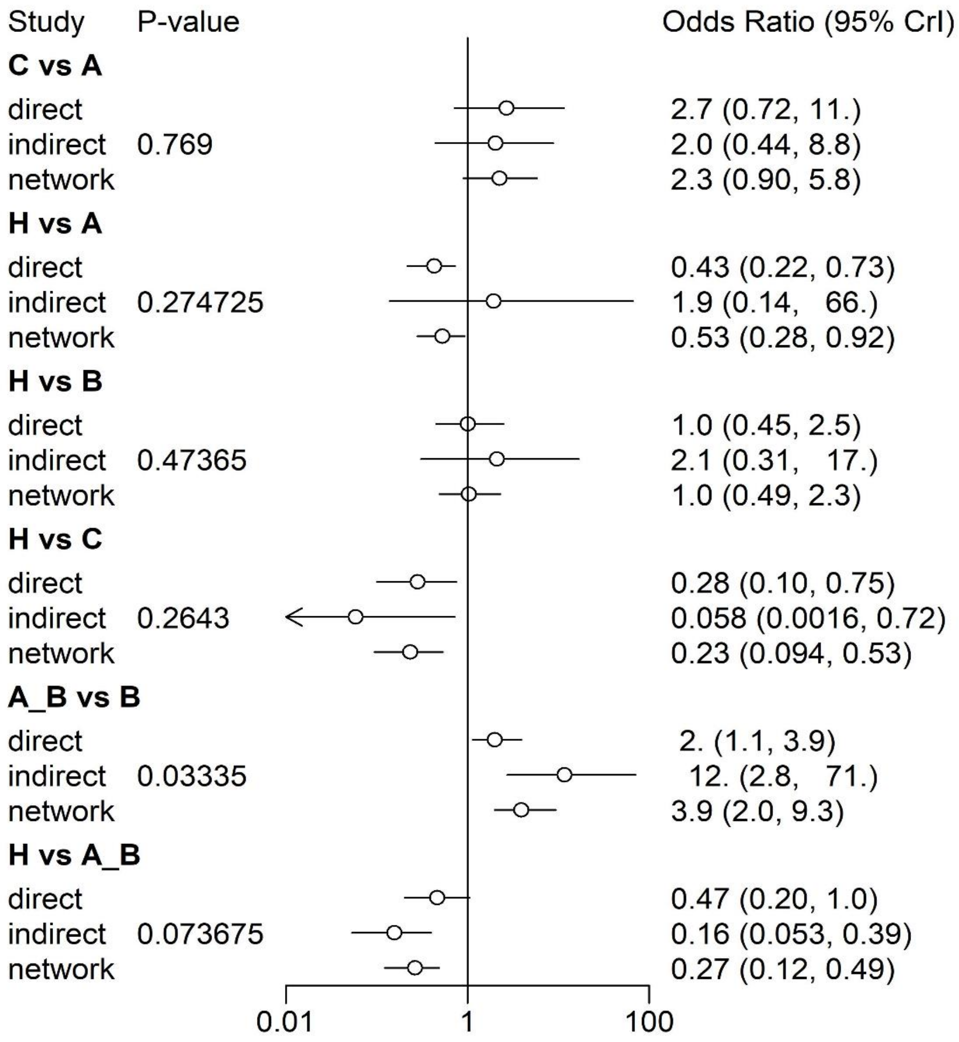

Figure S3B

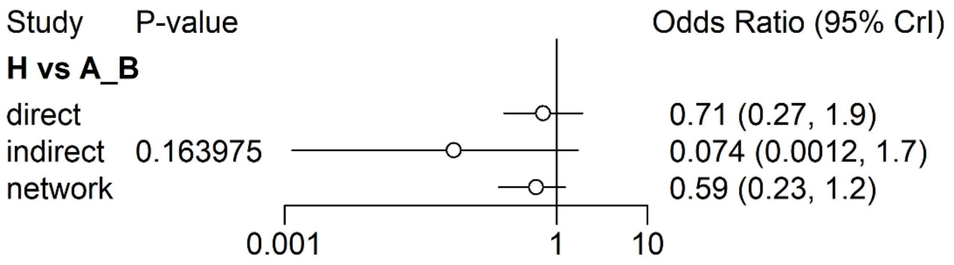

Figure S3C

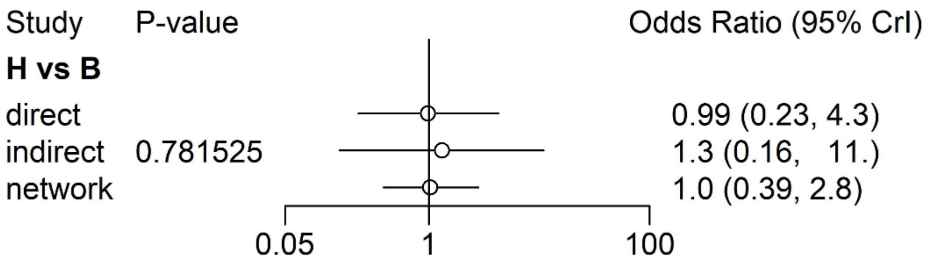

Figure S4

Figure S4A

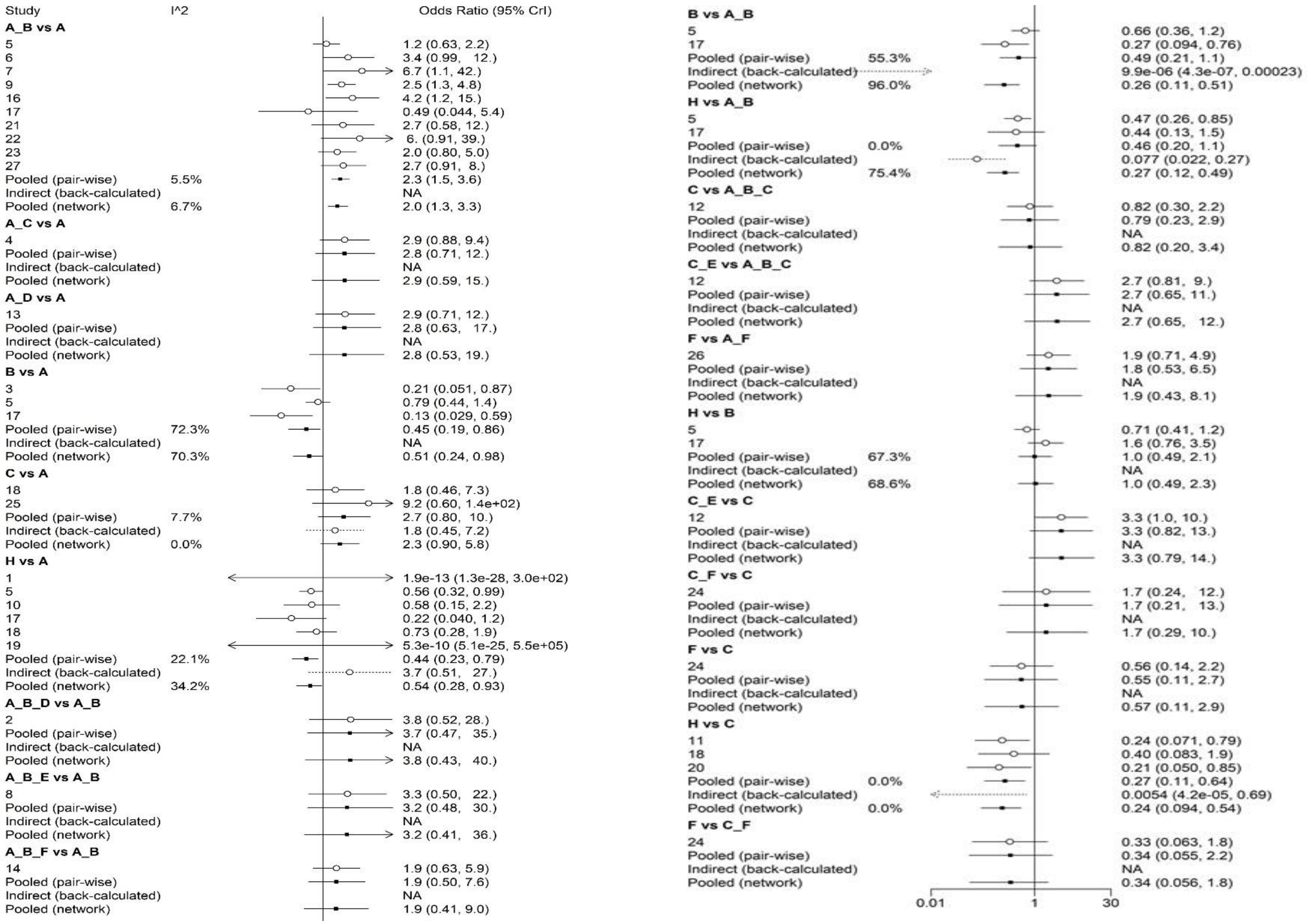

Figure S4B

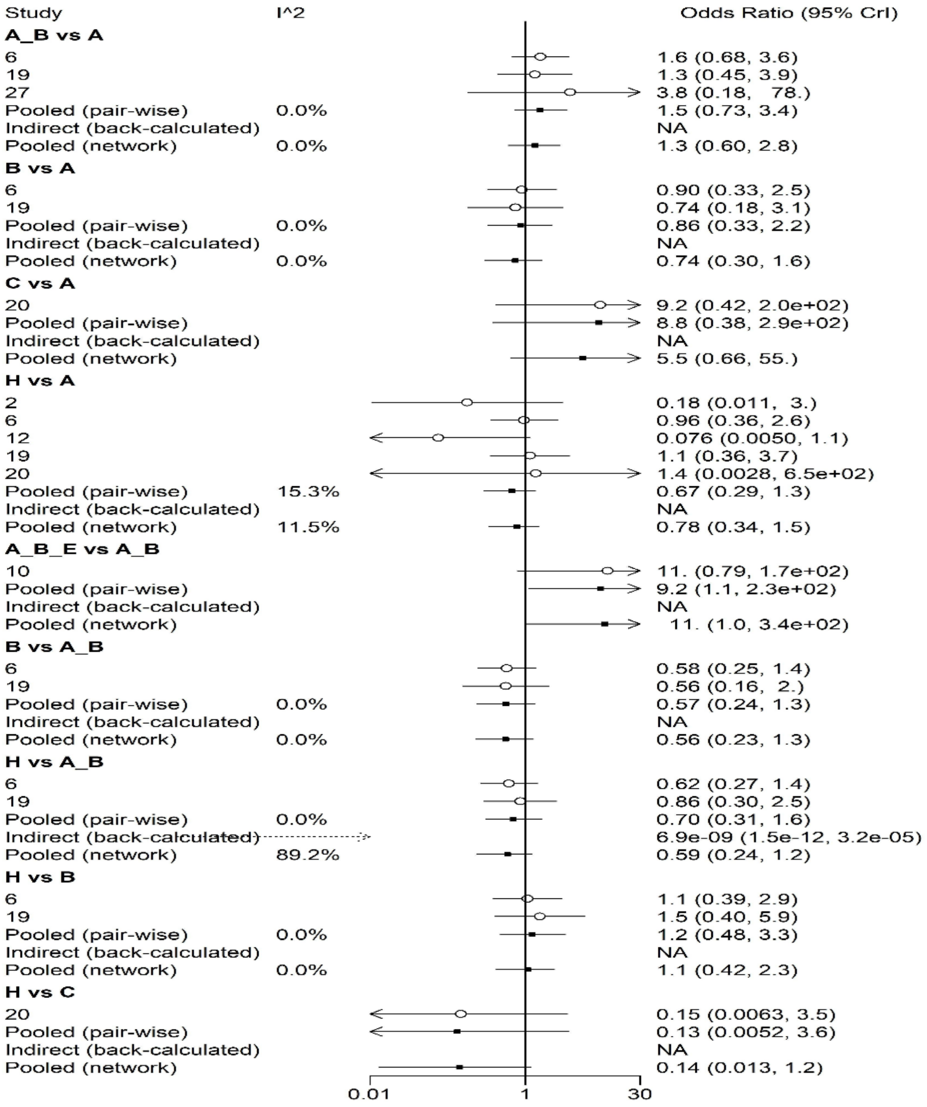
Study
I^2
Odds Ratio (95% CrI)
A_B vs A
6
19
27
Pooled (pair-wise) 0.0%
Indirect (back-calculated)
Pooled (network) 0.0%
1.6 (0.68, 3.6)
1.3 (0.45, 3.9)
3.8 (0.18, 78.)
1.5 (0.73, 3.4)
NA
1.3 (0.60, 2.8)
B vs A
6
19
Pooled (pair-wise) 0.0%
Indirect (back-calculated)
Pooled (network) 0.0%
0.90 (0.33, 2.5)
0.74 (0.18, 3.1)
0.86 (0.33, 2.2)
NA
0.74 (0.30, 1.6)
C vs A
20
Pooled (pair-wise)
Indirect (back-calculated)
Pooled (network)
9.2 (0.42, 2.0e+02)
8.8 (0.38, 2.9e+02)
NA
5.5 (0.66, 55.)
H vs A
2
6
12
19
20
Pooled (pair-wise) 15.3%
Indirect (back-calculated)
Pooled (network) 11.5%
0.18 (0.011, 3.)
0.96 (0.36, 2.6)
0.076 (0.0050, 1.1)
1.1 (0.36, 3.7)
1.4 (0.0028, 6.5e+02)
0.67 (0.29, 1.3)
NA
0.78 (0.34, 1.5)
A_B_E vs A_B
10
Pooled (pair-wise)
Indirect (back-calculated)
Pooled (network)
11. (0.79, 1.7e+02)
9.2 (1.1, 2.3e+02)
NA
11. (1.0, 3.4e+02)
B vs A_B
6
19
Pooled (pair-wise) 0.0%
Indirect (back-calculated)
Pooled (network) 0.0%
0.58 (0.25, 1.4)
0.56 (0.16, 2.)
0.57 (0.24, 1.3)
NA
0.56 (0.23, 1.3)
H vs A_B
6
19
Pooled (pair-wise) 0.0%
Indirect (back-calculated)
Pooled (network) 89.2%
0.62 (0.27, 1.4)
0.86 (0.30, 2.5)
0.70 (0.31, 1.6)
6.9e-09 (1.5e-12, 3.2e-05)
0.59 (0.24, 1.2)
H vs B
6
19
Pooled (pair-wise) 0.0%
Indirect (back-calculated)
Pooled (network) 0.0%
1.1 (0.39, 2.9)
1.5 (0.40, 5.9)
1.2 (0.48, 3.3)
NA
1.1 (0.42, 2.3)
H vs C
20
Pooled (pair-wise)
Indirect (back-calculated)
Pooled (network)
0.15 (0.0063, 3.5)
0.13 (0.0052, 3.6)
NA
0.14 (0.013, 1.2)
0.01
1
30

Figure S4C

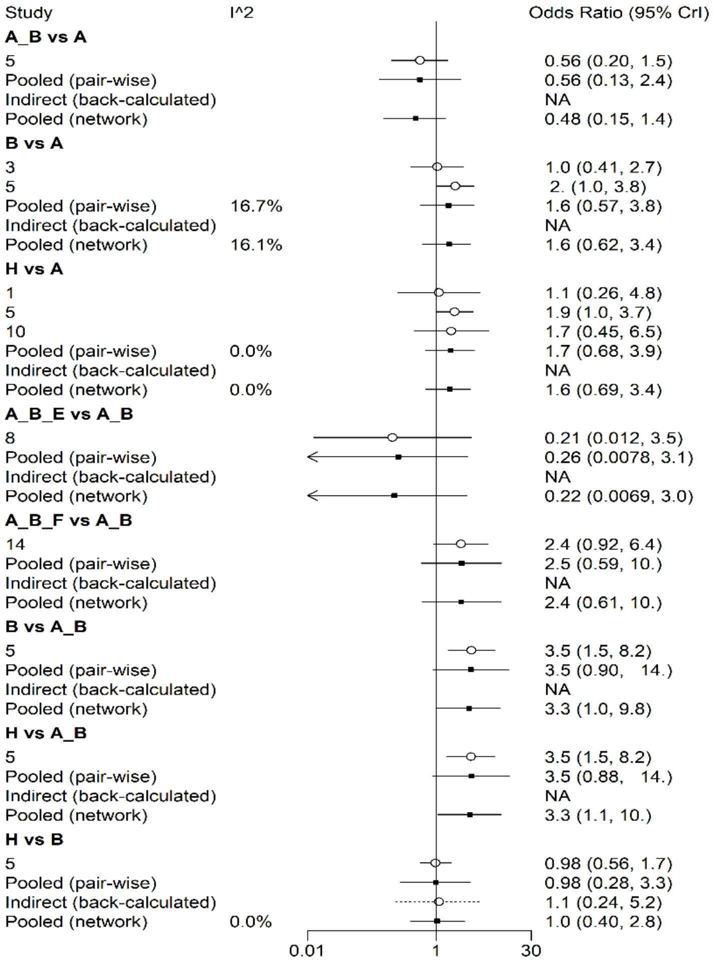
Study
I^2
Odds Ratio (95% CrI)
A_B vs A
5
0.56 (0.20, 1.5)
Pooled (pair-wise)
0.56 (0.13, 2.4)
Indirect (back-calculated)
NA
Pooled (network)
0.48 (0.15, 1.4)
B vs A
3
1.0 (0.41, 2.7)
5
2. (1.0, 3.8)
Pooled (pair-wise)
16.7%
1.6 (0.57, 3.8)
Indirect (back-calculated)
NA
Pooled (network)
16.1%
1.6 (0.62, 3.4)
H vs A
1
1.1 (0.26, 4.8)
5
1.9 (1.0, 3.7)
10
1.7 (0.45, 6.5)
Pooled (pair-wise)
0.0%
1.7 (0.68, 3.9)
Indirect (back-calculated)
NA
Pooled (network)
0.0%
1.6 (0.69, 3.4)
A_B_E vs A_B
8
0.21 (0.012, 3.5)
Pooled (pair-wise)
0.26 (0.0078, 3.1)
Indirect (back-calculated)
NA
Pooled (network)
0.22 (0.0069, 3.0)
A_B_F vs A_B
14
2.4 (0.92, 6.4)
Pooled (pair-wise)
2.5 (0.59, 10.)
Indirect (back-calculated)
NA
Pooled (network)
2.4 (0.61, 10.)
B vs A_B
5
3.5 (1.5, 8.2)
Pooled (pair-wise)
3.5 (0.90, 14.)
Indirect (back-calculated)
NA
Pooled (network)
3.3 (1.0, 9.8)
H vs A_B
5
3.5 (1.5, 8.2)
Pooled (pair-wise)
3.5 (0.88, 14.)
Indirect (back-calculated)
NA
Pooled (network)
3.3 (1.1, 10.)
H vs B
5
0.98 (0.56, 1.7)
Pooled (pair-wise)
0.98 (0.28, 3.3)
Indirect (back-calculated)
1.1 (0.24, 5.2)
Pooled (network)
0.0%
1.0 (0.40, 2.8)
0.01
1
30

Figure S5

Figure S5A (linear regression test, P=0.1175)

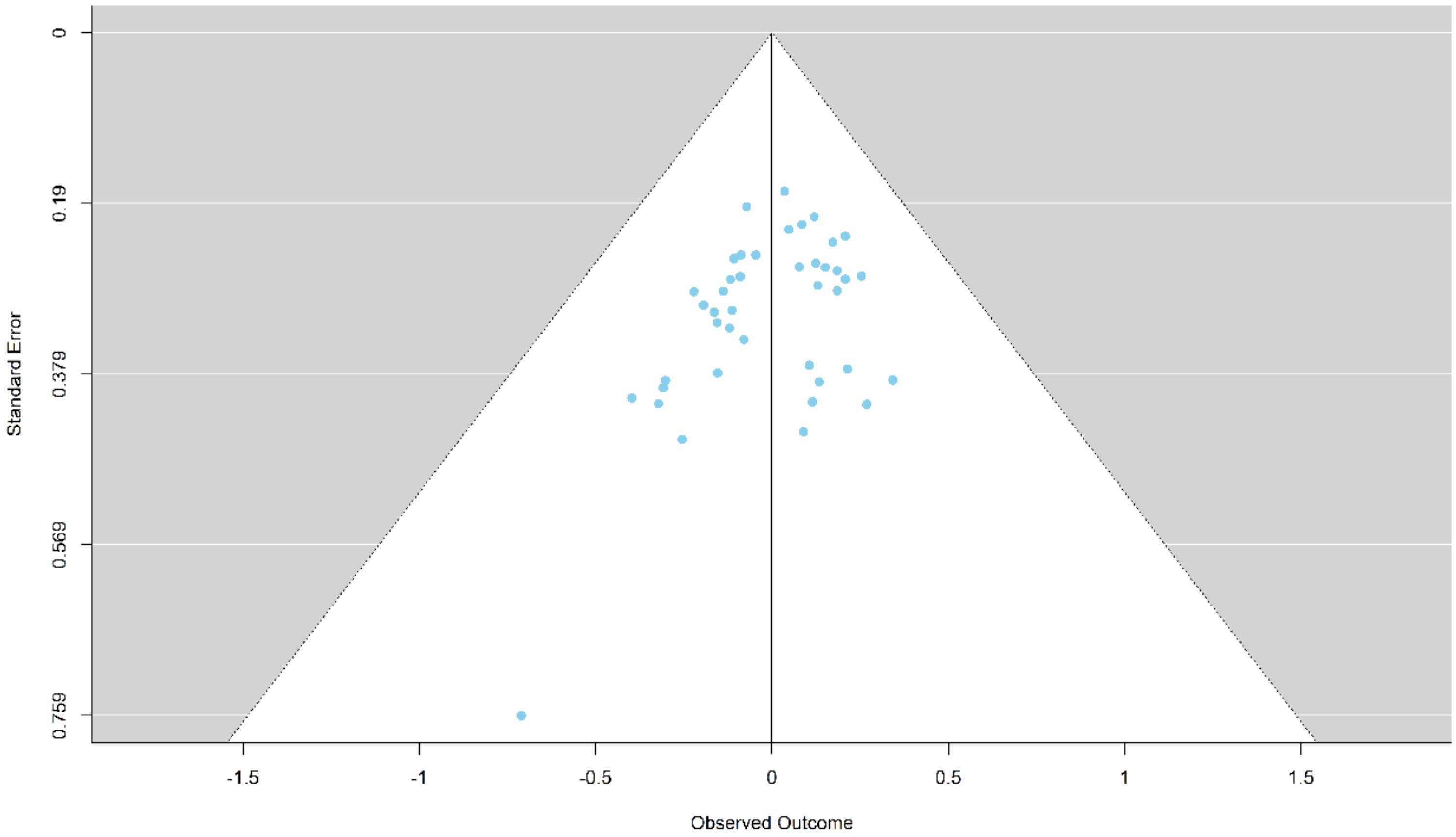

Figure S5B (linear regression test, P=0.2560)

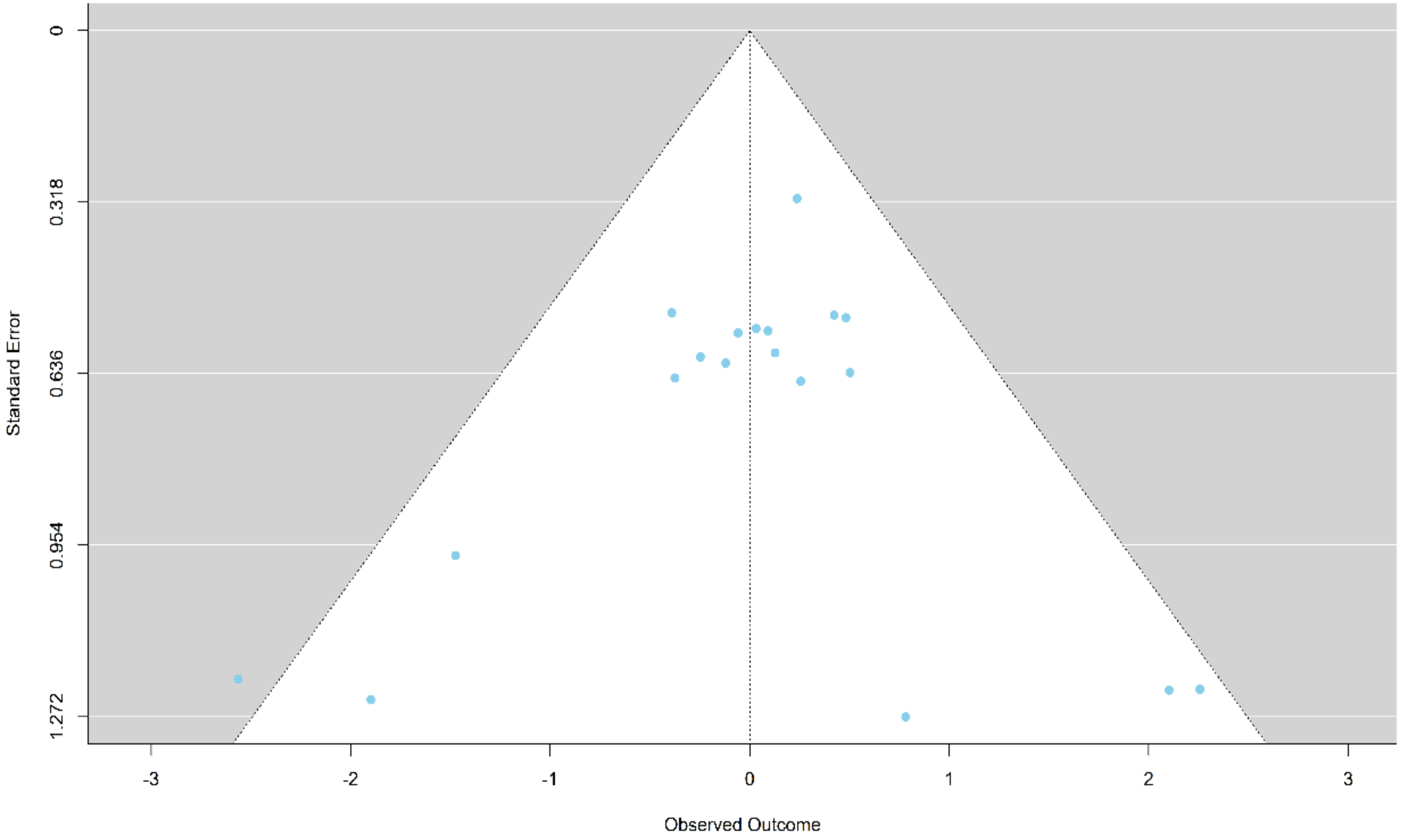

Figure S5C (linear regression test, P=0.7391)

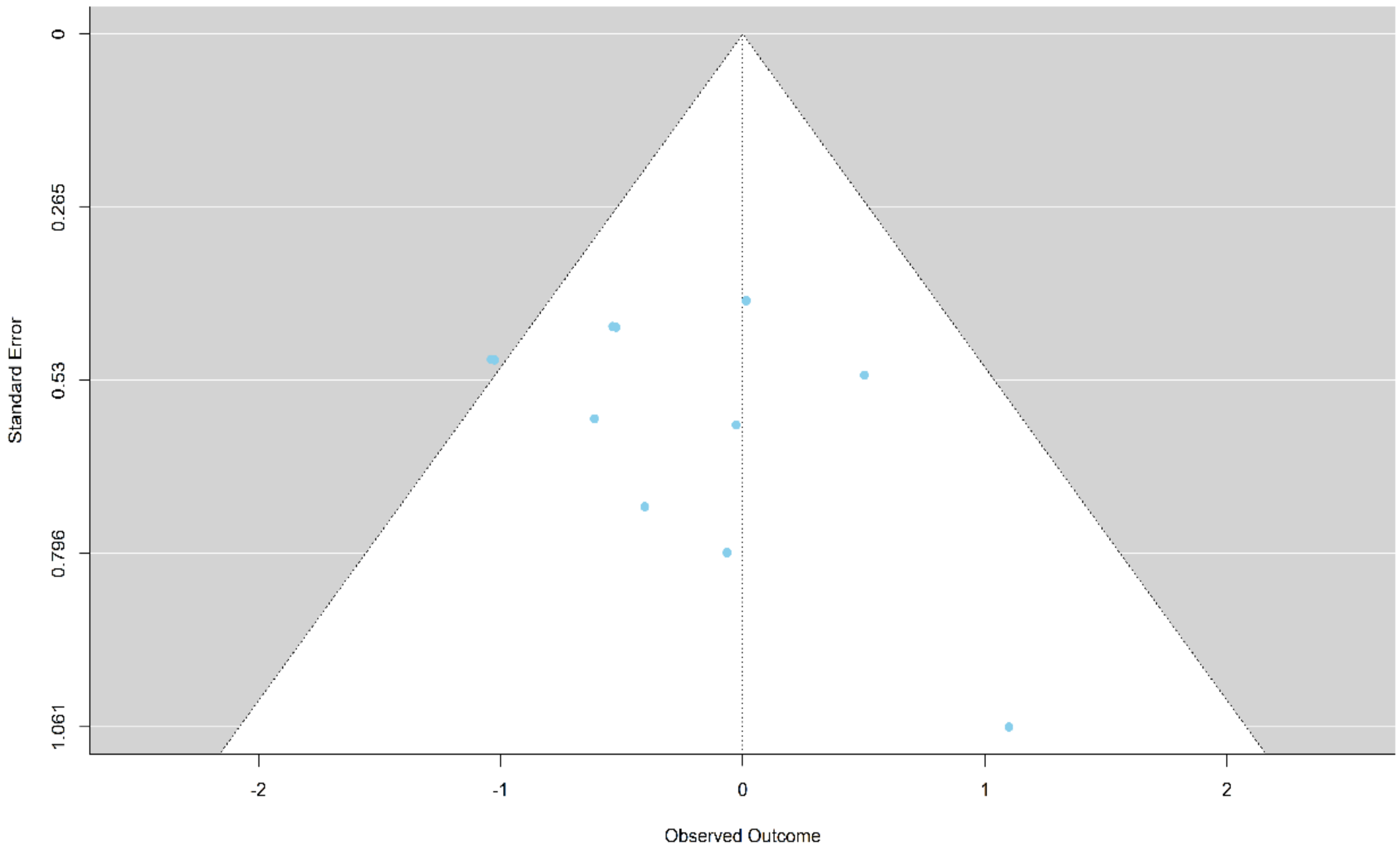

Figure S6

Figure S6A

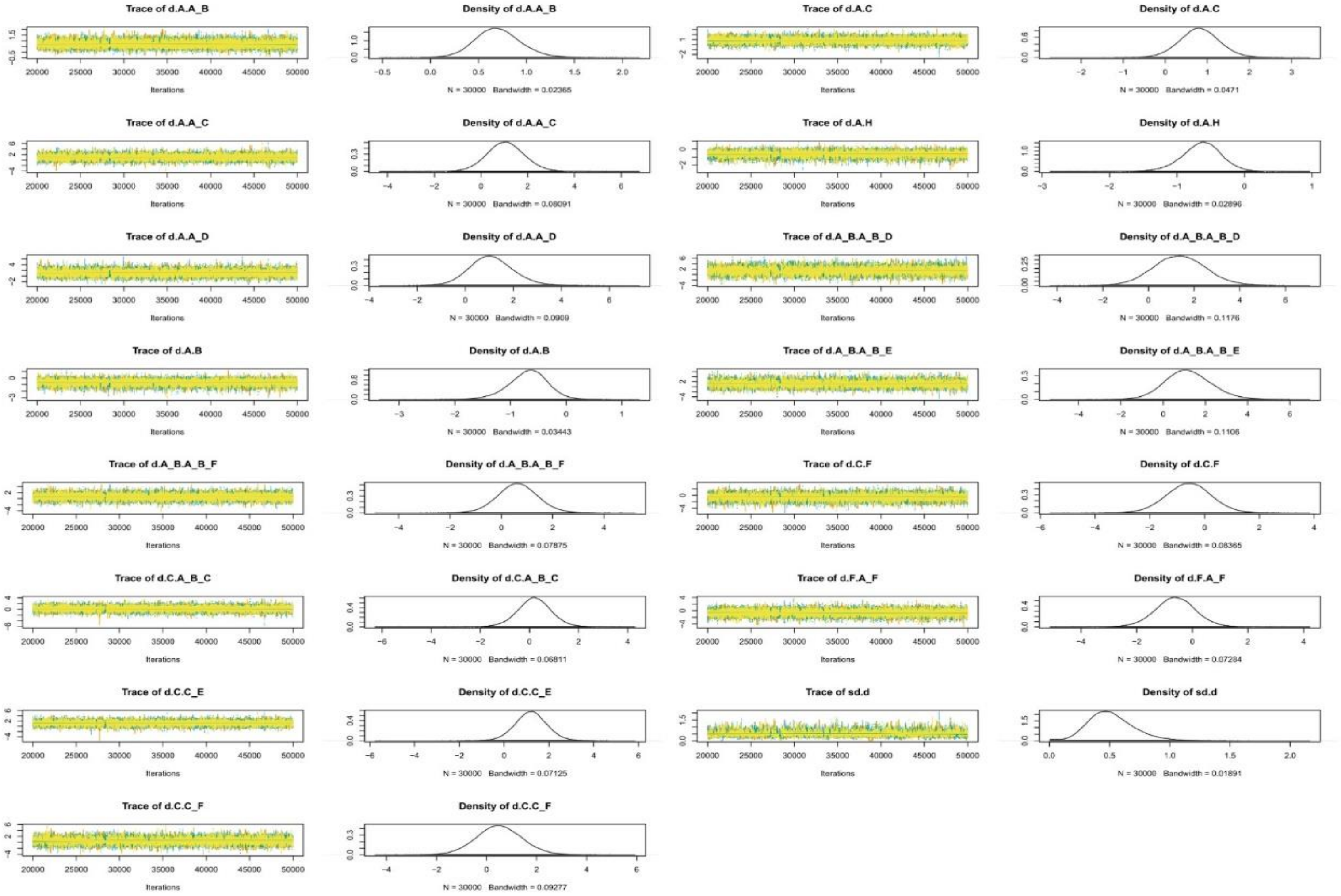

Figure S6B

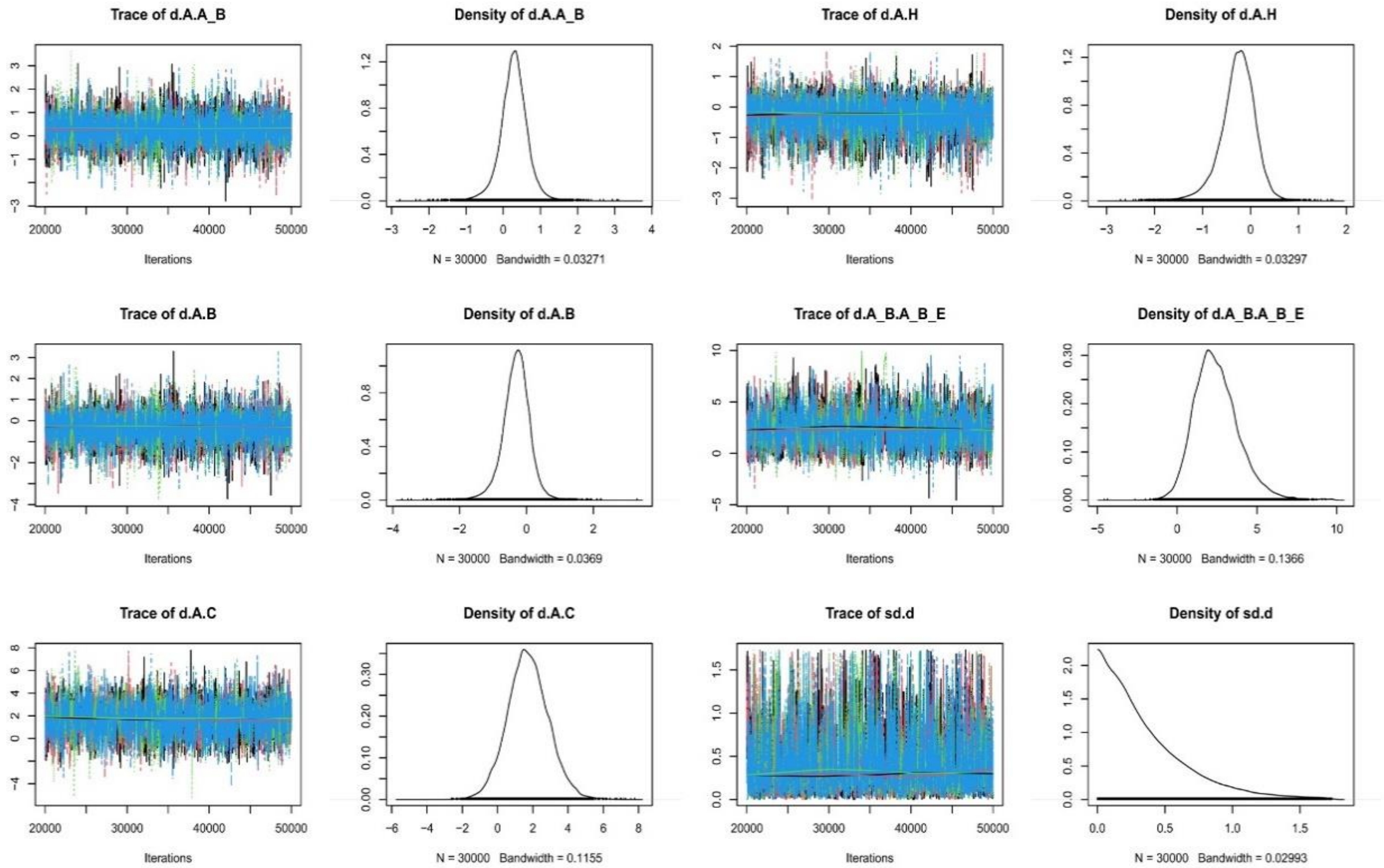
Trace of d.A.A_B
Iterations
Density of d.A.A_B
N = 30000 Bandwidth = 0.03271
Trace of d.A.H
Iterations
Density of d.A.H
N = 30000 Bandwidth = 0.03297
Trace of d.A.B
Iterations
Density of d.A.B
N = 30000 Bandwidth = 0.0369
Trace of d.A_B.A_B_E
Iterations
Density of d.A_B.A_B_E
N = 30000 Bandwidth = 0.1366
Trace of d.A.C
Iterations
Density of d.A.C
N = 30000 Bandwidth = 0.1155
Trace of sd.d
Iterations
Density of sd.d
N = 30000 Bandwidth = 0.02993

Figure S6C

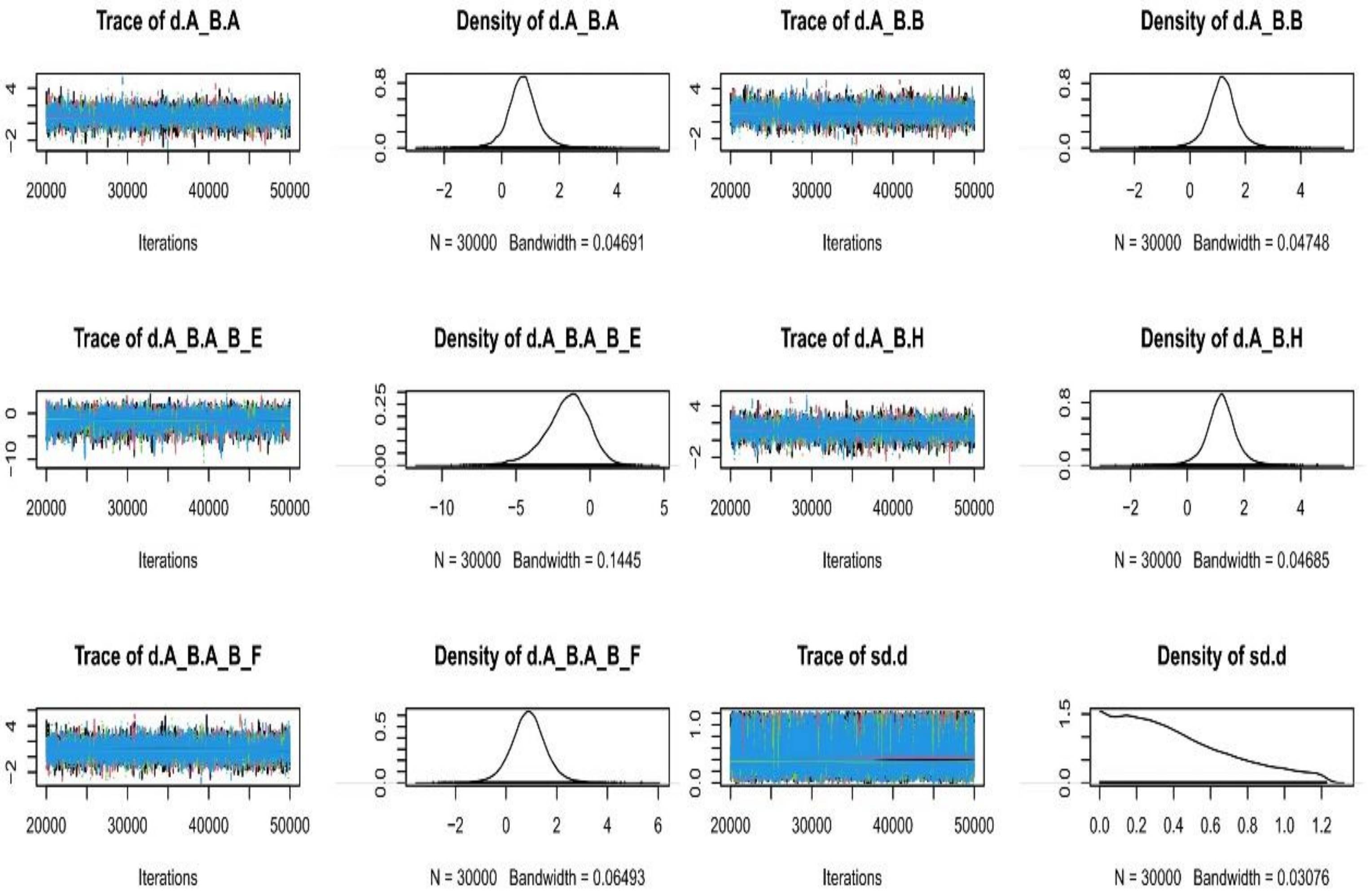
Trace of d.A_B.A
Iterations
Density of d.A_B.A
N = 30000 Bandwidth = 0.04691
Trace of d.A_B.B
Iterations
Density of d.A_B.B
N = 30000 Bandwidth = 0.04748
Trace of d.A_B.A_B_E
Iterations
Density of d.A_B.A_B_E
N = 30000 Bandwidth = 0.1445
Trace of d.A_B.H
Iterations
Density of d.A_B.H
N = 30000 Bandwidth = 0.04685
Trace of d.A_B.A_B_F
Iterations
Density of d.A_B.A_B_F
N = 30000 Bandwidth = 0.06493
Trace of sd.d
Iterations
Density of sd.d
N = 30000 Bandwidth = 0.03076

Figure S6D

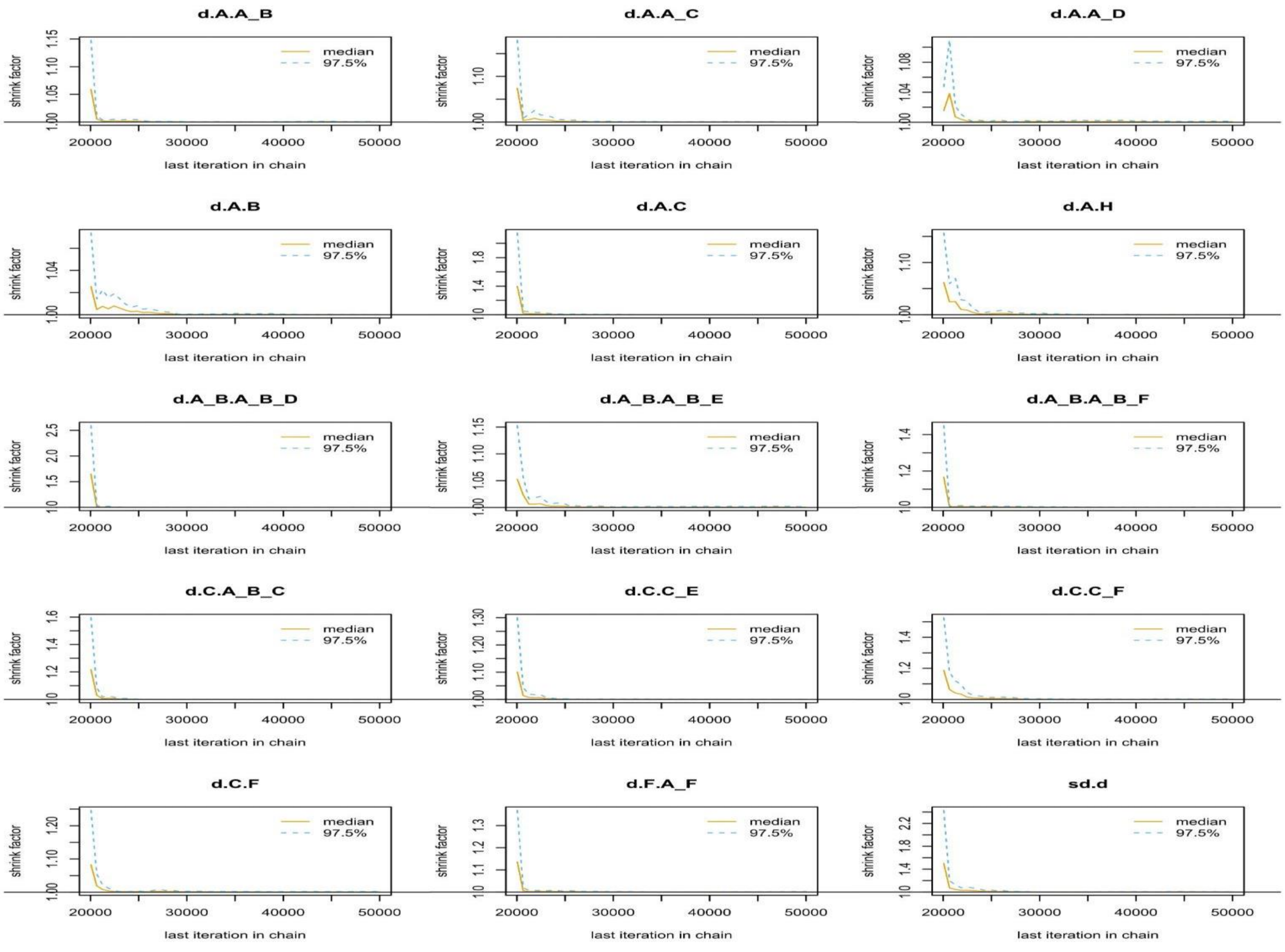
d.A.A_B
d.A.A_C
d.A.A_D
d.A.B
d.A.C
d.A.H
d.A_B.A_B_D
d.A_B.A_B_E
d.A_B.A_B_F
d.C.A_B_C
d.C.C_E
d.C.C_F
d.C.F
d.F.A_F
sd.d
median
97.5%
shrink factor
20000
30000
40000
50000
last iteration in chain

Figure S6E

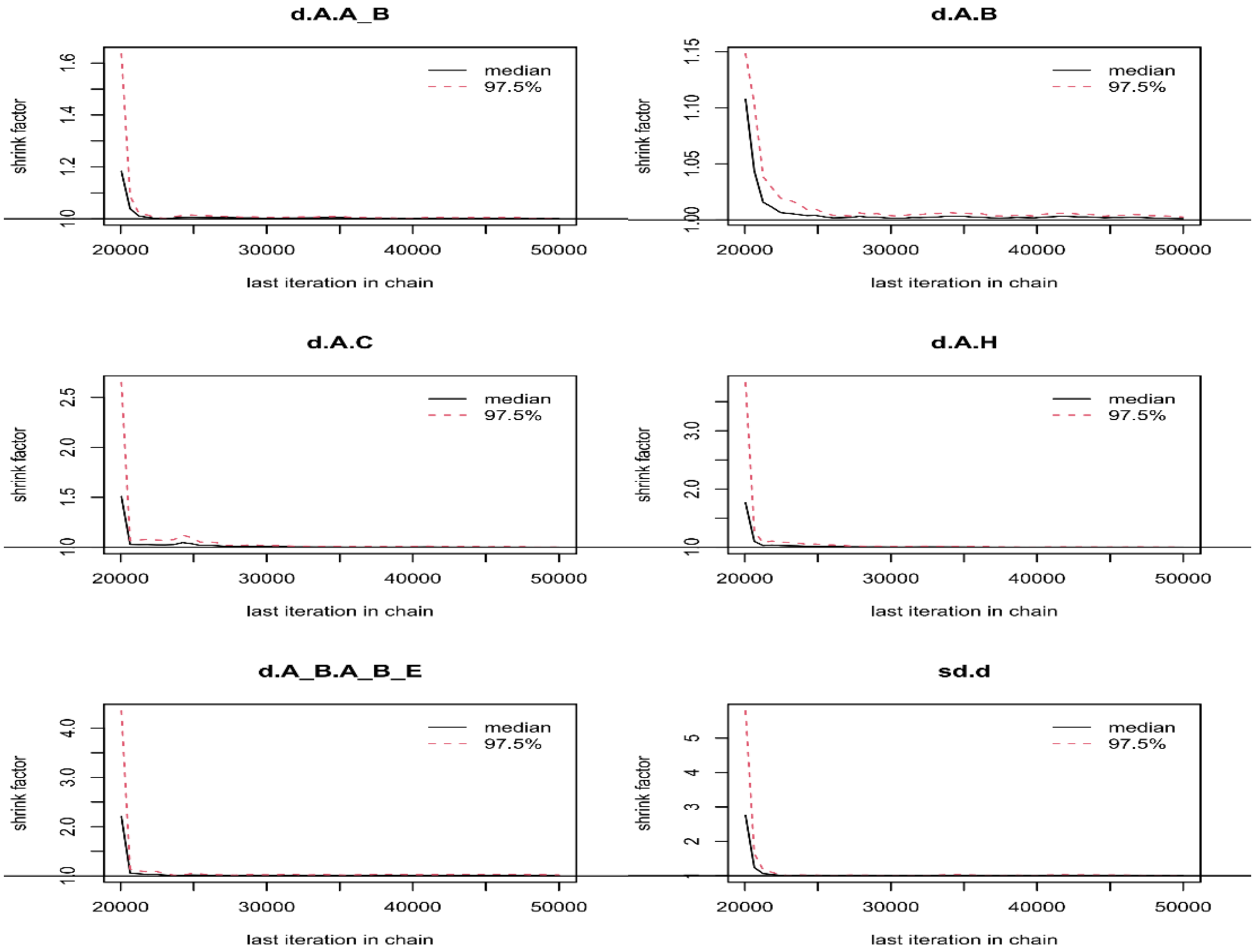
d.A.A_B
d.A.B
d.A.C
d.A.H
d.A_B.A_B_E
sd.d
median
97.5%
shrink factor
last iteration in chain
20000
30000
40000
50000
1.0
1.2
1.4
1.6
1.00
1.05
1.10
1.15
1.5
2.0
2.5
3.0
4.0
2
3
4
5

Figure S6F

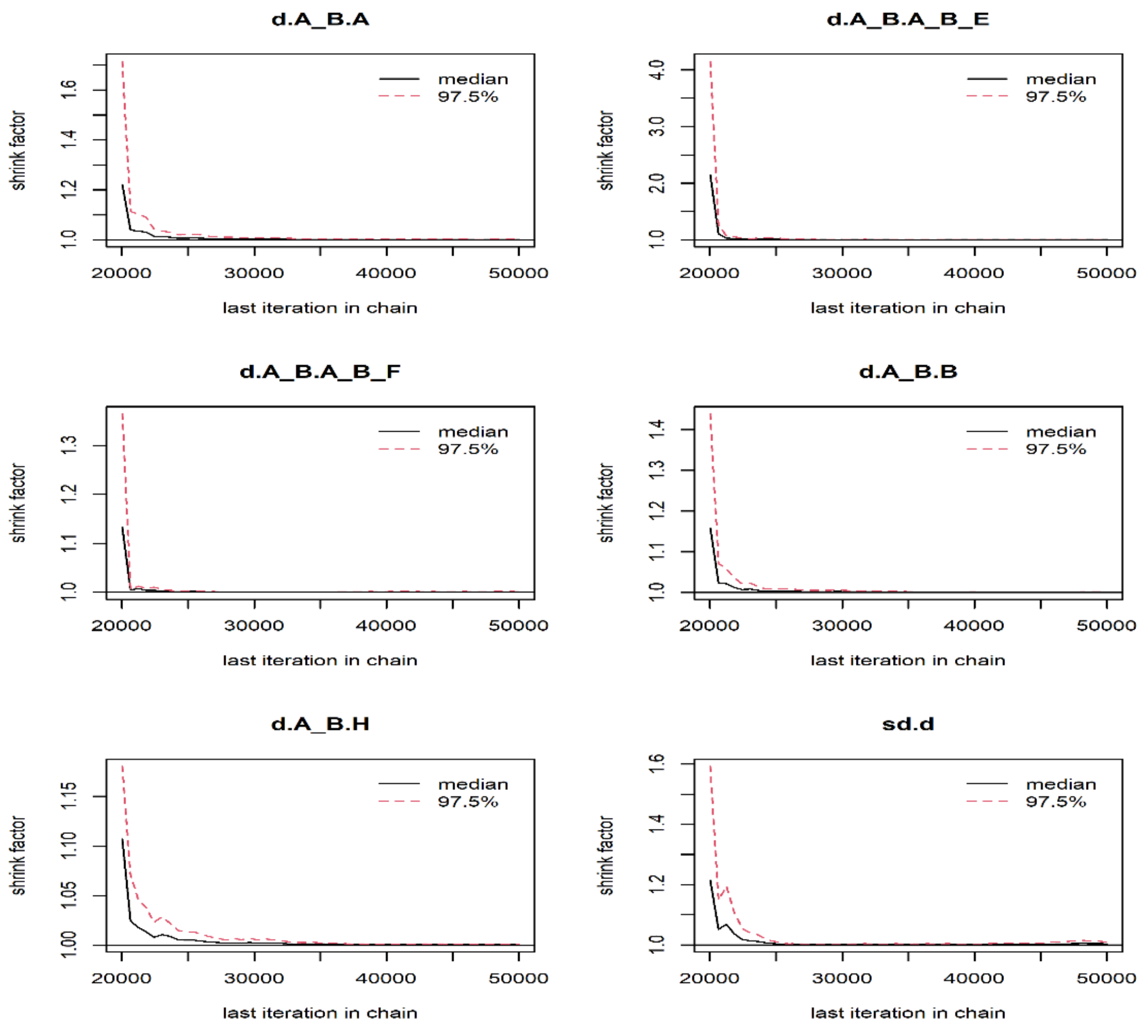
d.A_B.A
d.A_B.A_B_E
d.A_B.A_B_F
d.A_B.B
d.A_B.H
sd.d
median
97.5%
shrink factor
last iteration in chain
20000
30000
40000
50000
1.0
1.2
1.4
1.6
2.0
3.0
4.0
1.1
1.3
1.00
1.05
1.10
1.15

Figure S7

Figure S7A

A3+B	0.40 (0.10, 1.64)	0 (0, 6.21)	0 (0, 6.39)	0 (0, 6.13)	0 (0, 4.69)	0 (0, 4.08)	0 (0, 3.74)	0 (0, 2.96)	0 (0, 3.48)	0 (0, 2.25)	0 (0, 1.62)	0 (0, 1.12)	0 (0, 1.10)	0 (0, 1.08)	0 (0, 0.60)	0 (0, 0.64)	0 (0, 0.36)	0 (0, 0.34)
2.49 (0.61, 10.20)	A3	0 (0, 13.99)	0 (0, 14.78)	0 (0, 14.40)	0 (0, 10.57)	0 (0, 9.31)	0 (0, 8.72)	0 (0, 6.93)	0 (0, 8.16)	0 (0, 5.22)	0 (0, 3.84)	0 (0, 2.65)	0 (0, 2.56)	0 (0, 2.41)	0 (0, 1.38)	0 (0, 1.42)	0 (0, 0.83)	0 (0, 0.81)
> 100 (0.16, > 100)	> 100 (0.07, > 100)	C+E	1.01 (0.06, 15.91)	0.79 (0.04, 17.29)	0.66 (0.04, 14.77)	0.59 (0.04, 9.15)	0.56 (0.04, 7.58)	0.52 (0.06, 4.71)	0.52 (0.05, 5.91)	0.37 (0.08, 1.72)	0.30 (0.06, 1.41)	0.21 (0.03, 1.44)	0.20 (0.03, 1.40)	0.17 (0.02, 1.72)	0.11 (0.02, 0.70)	0.09 (0.01, 1.49)	0.07 (0.01, 0.39)	0.06 (0.01, 0.40)
> 100 (0.16, > 102)	> 100 (0.07, > 100)	0.99 (0.06, 15.73)	A1+B+F	0.78 (0.03, 21.70)	0.66 (0.030, 19.20)	0.59 (0.04, 8.91)	0.56 (0.04, 7.17)	0.53 (0.10, 2.69)	0.52 (0.03, 10.03)	0.37 (0.02, 5.57)	0.30 (0.03, 2.95)	0.21 (0.02, 2.07)	0.20 (0.03, 1.32)	0.17 (0.01, 3.01)	0.11 (0.01, 1.03)	0.09 (0.003, 2.42)	0.07 (0.01, 0.58)	0.06 (0.01, 0.55)
> 100 (0.16, > 100)	> 100 (0.07, > 100)	1.26 (0.06, 24.30)	1.28 (0.05, 31.46)	A2+B+D	0.85 (0.03, 22.88)	0.75 (0.03, 17.82)	0.72 (0.03, 15.85)	0.66 (0.04, 10.40)	0.65 (0.03, 15.39)	0.47 (0.02, 8.64)	0.39 (0.03, 4.81)	0.26 (0.02, 2.60)	0.26 (0.02, 3.34)	0.22 (0.01, 4.7)	0.14 (0.01, 1.47)	0.12 (0.003, 3.61)	0.09 (0.01, 0.94)	0.08 (0.01, 0.86)
> 100 (0.21, > 100)	> 100 (0.10, > 100)	1.51 (0.07, 25.66)	1.51 (0.05, 34.05)	1.17 (0.04, 29.90)	A2+B+E	0.89 (0.03, 19.74)	0.85 (0.03, 16.90)	0.79 (0.04, 10.92)	0.77 (0.03, 16.26)	0.56 (0.03, 9.21)	0.46 (0.03, 4.98)	0.32 (0.03, 2.53)	0.30 (0.02, 3.40)	0.26 (0.01, 4.89)	0.17 (0.01, 1.45)	0.14 (0.004, 3.79)	0.10 (0.01, 0.94)	0.09 (0.01, 0.87)
> 100 (0.25, > 100)	> 100 (0.11, > 100)	1.70 (0.11, 24.72)	1.71 (0.11, 24.12)	1.34 (0.06, 35.24)	1.13 (0.05, 29.88)	A1+D	0.96 (0.07, 11.32)	0.90 (0.10, 6.93)	0.88 (0.05, 16.40)	0.63 (0.04, 8.82)	0.52 (0.05, 4.56)	0.36 (0.04, 3.21)	0.35 (0.05, 2.02)	0.30 (0.02, 4.77)	0.19 (0.02, 1.61)	0.16 (0.01, 3.87)	0.12 (0.01, 0.90)	0.11 (0.01, 0.86)
> 100 (0.27, > 100)	> 100 (0.12, > 100)	1.78 (0.13, 24.08)	1.77 (0.14, 23.44)	1.40 (0.06, 34.68)	1.18 (0.06, 29.52)	1.04 (0.09, 13.73)	A1+C	0.93 (0.13, 6.62)	0.92 (0.06, 15.85)	0.66 (0.05, 8.64)	0.54 (0.07, 4.41)	0.37 (0.04, 3.04)	0.36 (0.06, 1.89)	0.31 (0.02, 4.64)	0.20 (0.02, 1.52)	0.17 (0.01, 3.88)	0.12 (0.02, 0.85)	0.11 (0.01, 0.81)
> 100 (0.34, > 100)	> 100 (0.14, > 100)	1.92 (0.21, 17.40)	1.90 (0.37, 10.20)	1.50 (0.10, 26.55)	1.27 (0.09, 23.19)	1.11 (0.14, 9.85)	1.08 (0.15, 7.76)	A1+B	0.99 (0.09, 12.00)	0.71 (0.08, 6.23)	0.58 (0.12, 2.80)	0.40 (0.08, 1.96)	0.38 (0.14, 0.99)	0.33 (0.03, 3.45)	0.21 (0.04, 0.97)	0.18 (0.01, 2.92)	0.13 (0.03, 0.53)	0.12 (0.02, 0.52)
> 100 (0.29, > 100)	> 100 (0.12, > 100)	1.93 (0.17, 21.67)	1.93 (0.10, 38.51)	1.53 (0.07, 38.90)	1.30 (0.06, 32.27)	1.13 (0.06, 21.01)	1.09 (0.06, 18.09)	1.01 (0.08, 11.65)	C+F	0.715 (0.06, 7.75)	0.59 (0.09, 3.71)	0.40 (0.04, 3.56)	0.39 (0.04, 3.56)	0.34 (0.05, 2.01)	0.22 (0.02, 1.77)	0.18 (0.02, 1.92)	0.13 (0.02, 0.99)	0.12 (0.01, 1.03)
> 100 (0.44, > 100)	> 100 (0.19, > 100)	2.69 (0.58, 12.86)	2.70 (0.18, 42.82)	2.11 (0.12, 45.46)	1.79 (0.11, 39.80)	1.58 (0.11, 24.29)	1.52 (0.12, 20.32)	1.41 (0.16, 12.43)	1.40 (0.13, 15.69)	A1+B+C	0.82 (0.18, 3.66)	0.56 (0.09, 3.74)	0.54 (0.08, 3.67)	0.47 (0.05, 4.57)	0.30 (0.05, 1.84)	0.25 (0.02, 3.90)	0.18 (0.03, 1.020)	0.17 (0.02, 1.06)
> 100 (0.62, > 100)	> 100 (0.26, > 100)	3.285 (0.71, 15.39)	3.29 (0.34, 33.63)	2.58 (0.21, 38.11)	2.17 (0.20, 31.86)	1.92 (0.22, 19.33)	1.85 (0.23, 15.11)	1.72 (0.36, 8.50)	1.70 (0.27, 11.64)	1.22 (0.27, 5.43)	C	0.68 (0.22, 2.19)	0.66 (0.19, 2.24)	0.57 (0.10, 3.16)	0.37 (0.12, 1.04)	0.31 (0.03, 3.10)	0.22 (0.09, 0.54)	0.21 (0.06, 0.62)
> 100 (0.90, > 100)	> 100 (0.38, > 100)	4.82 (0.69, 32.25)	4.83 (0.48, 48.00)	3.78 (0.39, 42.50)	3.16 (0.40, 35.96)	2.82 (0.31, 28.53)	2.71 (0.33, 22.47)	2.51 (0.51, 12.30)	2.49 (0.28, 23.20)	1.79 (0.27, 11.62)	1.47 (0.46, 4.53)	A2+B	0.96 (0.27, 3.32)	0.84 (0.10, 6.48)	0.54 (0.26, 0.98)	0.45 (0.03, 5.88)	0.33 (0.13, 0.70)	0.30 (0.11, 0.67)
> 100 (0.91, > 100)	> 100 (0.39, > 100)	5.00 (0.71, 36.73)	4.99 (0.76, 35.50)	3.92 (0.30, 59.67)	3.30 (0.30, 51.97)	2.90 (0.50, 21.01)	2.81 (0.53, 15.91)	2.61 (1.10, 7.10)	2.60 (0.31, 28.87)	1.86 (0.27, 13.11)	1.52 (0.45, 5.35)	1.04 (0.30, 3.73)	A1	0.87 (0.10, 7.29)	0.56 (0.16, 1.84)	0.47 (0.03, 6.51)	0.34 (0.11, 0.96)	0.31 (0.09, 0.95)
> 100 (0.93, > 100)	> 100 (0.42, > 100)	5.77 (0.58, 59.60)	5.75 (0.33, 105.45)	4.55 (0.21, 110.28)	3.86 (0.21, 91.33)	3.38 (0.21, 60.72)	3.25 (0.22, 49.24)	3.02 (0.29, 31.73)	2.96 (0.50, 19.75)	2.14 (0.22, 21.21)	1.75 (0.32, 9.83)	1.19 (0.15, 9.77)	1.15 (0.14, 9.61)	F	0.64 (0.08, 4.80)	0.54 (0.11, 2.53)	0.39 (0.06, 2.72)	0.36 (0.04, 2.82)
> 100 (1.68, > 100)	> 100 (0.72, > 100)	8.97 (1.43, 60.38)	8.97 (0.97, 92.48)	7.10 (0.68, 88.72)	5.93 (0.69, 76.70)	5.25 (0.62, 54.03)	5.04 (0.66, 43.07)	4.68 (1.03, 23.54)	4.64 (0.56, 43.41)	3.32 (0.54, 21.59)	2.73 (0.96, 8.26)	1.86 (1.02, 3.84)	1.79 (0.55, 6.23)	1.56 (0.21, 12.20)	A2	0.84 (0.07, 11.13)	0.61 (0.29, 1.29)	0.56 (0.23, 1.27)
> 100 (1.56, > 100)	> 100 (0.70, > 100)	10.63 (0.67, 178.18)	10.69 (0.41, 292.75)	8.49 (0.28, 295.90)	7.14 (0.26, 247.01)	6.30 (0.26, 166.71)	8.26 (1.18, 64.65)	5.59 (0.34, 97.01)	5.51 (0.52, 63.81)	3.97 (0.26, 63.69)	3.25 (0.32, 33.59)	2.22 (0.17, 30.69)	2.13 (0.15, 29.81)	1.85 (0.40, 8.84)	1.19 (0.09, 15.25)	A2+F	0.72 (0.06, 8.77)	0.67 (0.05, 8.70)
> 100 (2.82, > 100)	> 100 (1.21, > 100)	14.72 (2.60, 91.03)	14.69 (1.72, 142.59)	11.58 (1.07, 151.72)	9.74 (1.06, 130.83)	8.60 (1.11, 82.87)	8.26 (1.18, 64.65)	7.66 (1.88, 34.65)	7.59 (1.01, 65.78)	5.45 (0.98, 32.49)	4.46 (1.87, 11.54)	3.03 (1.43, 7.48)	2.93 (1.04, 8.89)	2.55 (0.37, 18.31)	1.64 (0.78, 3.48)	1.38 (0.11, 17.12)	H	0.92 (0.38, 2.08)
> 100 (2.98, > 100)	> 100 (1.24, > 100)	15.99 (2.50, 115.73)	15.98 (1.81, 166.26)	12.66 (1.17, 170.00)	10.59 (1.15, 148.38)	9.32 (1.17, 96.59)	9.00 (1.24, 75.80)	8.36 (1.94, 41.55)	8.31 (0.97, 82.83)	5.91 (0.94, 41.91)	4.86 (1.62, 16.67)	3.29 (1.50, 8.83)	3.19 (1.05, 10.88)	2.78 (0.36, 23.37)	1.78 (0.79, 4.30)	1.50 (0.12, 20.91)	1.08 (0.48, 2.60)	B

Figure S7B

A+B1+D	0.85 (0.04, 17.58)	0.84 (0.05, 11.41)	0.51 (0.04, 6.14)	0.44 (0.02, 8.65)	0.33 (0.02, 5.42)	0.32 (0.02, 4.45)	0.31 (0.02, 4.15)	0.27 (0.03, 2.09)	0.26 (0.02, 2.63)	0.26 (0.02, 2.63)	0.18 (0.02, 1.79)	0.14 (0.01, 2.29)	0.12 (0.01, 1.00)	0.10 (0.01, 0.98)	0.08 (0.003, 1.58)	0.06 (0.01, 0.56)	0.03 (0.003, 0.32)
1.18 (0.057, 23.82)	A+B1+E	0.97 (0.06, 11.73)	0.59 (0.04, 6.75)	0.51 (0.03, 8.41)	0.39 (0.02, 5.71)	0.38 (0.02, 4.69)	0.36 (0.02, 4.39)	0.31 (0.03, 2.16)	0.30 (0.03, 2.68)	0.29 (0.03, 2.64)	0.21 (0.02, 1.83)	0.17 (0.01, 2.35)	0.13 (0.01, 1.03)	0.11 (0.01, 1.02)	0.09 (0.004, 1.68)	0.07 (0.01, 0.57)	0.04 (0.003, 0.33)
1.20 (0.09, 20.77)	1.04 (0.09, 15.99)	C+E	0.62 (0.07, 5.15)	0.54 (0.06, 4.30)	0.40 (0.05, 4.05)	0.40 (0.05, 3.08)	0.38 (0.10, 1.27)	0.32 (0.06, 1.61)	0.31 (0.06, 1.67)	0.31 (0.09, 1.04)	0.22 (0.05, 1.11)	0.18 (0.02, 1.17)	0.14 (0.03, 0.60)	0.12 (0.02, 0.61)	0.10 (0.01, 0.92)	0.08 (0.02, 0.31)	0.04 (0.007, 0.20)
1.96 (0.16, 28.38)	1.70 (0.15, 22.82)	1.62 (0.19, 13.77)	A+B1+F	0.88 (0.07, 9.80)	0.66 (0.06, 6.65)	0.65 (0.08, 5.20)	0.61 (0.07, 4.93)	0.53 (0.13, 2.02)	0.51 (0.09, 2.75)	0.50 (0.09, 2.82)	0.36 (0.07, 1.92)	0.29 (0.03, 2.67)	0.23 (0.05, 1.01)	0.20 (0.03, 1.06)	0.15 (0.01, 2.06)	0.12 (0.02, 0.57)	0.07 (0.01, 0.34)
2.26 (0.12, 49.16)	1.96 (0.12, 39.24)	1.84 (0.23, 16.16)	1.14 (0.10, 14.08)	C+F	0.77 (0.06, 10.30)	0.75 (0.07, 8.29)	0.70 (0.09, 5.62)	0.60 (0.08, 4.55)	0.58 (0.07, 4.64)	0.58 (0.10, 3.19)	0.41 (0.06, 3.13)	0.33 (0.06, 1.64)	0.26 (0.04, 1.73)	0.22 (0.03, 1.69)	0.17 (0.02, 1.35)	0.14 (0.02, 0.90)	0.08 (0.01, 0.58)
3.02 (0.19, 54.95)	2.60 (0.18, 42.44)	2.52 (0.25, 21.45)	1.52 (0.15, 15.57)	1.30 (0.10, 16.17)	A+D	0.99 (0.10, 8.36)	0.94 (0.10, 7.69)	0.81 (0.12, 4.40)	0.79 (0.11, 4.33)	0.78 (0.11, 4.36)	0.56 (0.09, 3.05)	0.44 (0.04, 4.30)	0.35 (0.06, 1.59)	0.30 (0.04, 1.68)	0.24 (0.01, 3.20)	0.19 (0.03, 0.92)	0.10 (0.02, 0.57)
3.09 (0.23, 49.48)	2.62 (0.21, 41.69)	2.53 (0.32, 19.93)	1.55 (0.19, 13.10)	1.34 (0.12, 15.21)	1.01 (0.12, 9.70)	A+C	0.95 (0.12, 7.17)	0.81 (0.16, 3.94)	0.80 (0.14, 3.99)	0.78 (0.15, 4.29)	0.55 (0.12, 2.77)	0.45 (0.05, 4.01)	0.35 (0.08, 1.45)	0.30 (0.06, 1.55)	0.24 (0.02, 3.01)	0.19 (0.04, 0.84)	0.10 (0.02, 0.51)
3.19 (0.24, 51.81)	2.79 (0.23, 42.42)	2.63 (0.79, 9.71)	1.64 (0.20, 13.63)	1.42 (0.18, 11.38)	1.07 (0.13, 10.56)	1.05 (0.14, 8.20)	A+B1+C	0.85 (0.18, 4.21)	0.81 (0.16, 4.35)	0.82 (0.25, 2.75)	0.58 (0.13, 2.95)	0.46 (0.07, 3.11)	0.37 (0.09, 1.57)	0.31 (0.06, 1.59)	0.25 (0.03, 2.45)	0.20 (0.05, 0.81)	0.11 (0.02, 0.53)
3.70 (0.48, 39.93)	3.21 (0.46, 29.87)	3.11 (0.62, 16.01)	1.89 (0.50, 7.95)	1.66 (0.22, 12.63)	1.24 (0.23, 8.37)	1.23 (0.25, 6.17)	1.18 (0.24, 5.55)	A+B1	0.97 (0.33, 2.72)	0.97 (0.33, 2.73)	0.68 (0.28, 1.78)	0.55 (0.09, 3.21)	0.43 (0.21, 0.84)	0.37 (0.13, 1.03)	0.29 (0.03, 2.60)	0.23 (0.10, 0.50)	0.13 (0.05, 0.31)
3.85 (0.38, 51.06)	3.30 (0.37, 38.67)	3.22 (0.60, 18.11)	1.95 (0.36, 11.79)	1.73 (0.22, 13.60)	1.27 (0.23, 9.09)	1.26 (0.25, 6.93)	1.23 (0.23, 6.33)	1.03 (0.37, 3.06)	A+B3	0.99 (0.32, 3.23)	0.70 (0.27, 2.15)	0.57 (0.08, 3.55)	0.44 (0.20, 1.00)	0.38 (0.12, 1.22)	0.30 (0.03, 2.85)	0.24 (0.09, 0.60)	0.13 (0.04, 0.40)
3.89 (0.38, 50.75)	3.41 (0.38, 38.44)	3.20 (0.97, 11.74)	2.00 (0.36, 11.72)	1.71 (0.31, 9.95)	1.28 (0.23, 9.21)	1.28 (0.23, 6.80)	1.22 (0.36, 4.00)	1.04 (0.37, 3.02)	1.01 (0.31, 3.18)	C	0.71 (0.27, 2.02)	0.57 (0.13, 2.45)	0.45 (0.19, 1.01)	0.38 (0.13, 1.15)	0.31 (0.04, 2.11)	0.24 (0.10, 0.51)	0.13 (0.04, 0.38)
5.47 (0.56, 65.72)	4.72 (0.55, 50.93)	4.55 (0.90, 22.26)	2.78 (0.52, 15.25)	2.45 (0.32, 17.61)	1.80 (0.33, 11.54)	1.80 (0.36, 8.58)	1.73 (0.34, 7.72)	1.47 (0.56, 3.58)	1.43 (0.46, 3.74)	1.41 (0.50, 3.72)	A+B2	0.81 (0.13, 4.44)	0.63 (0.30, 1.12)	0.55 (0.20, 1.21)	0.43 (0.04, 3.55)	0.35 (0.14, 0.64)	0.19 (0.06, 0.46)
6.92 (0.44, 130.71)	5.90 (0.43, 103.46)	5.63 (0.86, 41.50)	3.48 (0.38, 35.86)	3.03 (0.61, 16.24)	2.26 (0.23, 27.08)	2.23 (0.25, 21.07)	2.17 (0.32, 14.38)	1.81 (0.31, 11.55)	1.77 (0.28, 11.88)	1.76 (0.41, 7.97)	1.23 (0.23, 7.92)	F	0.78 (0.15, 4.36)	0.67 (0.11, 4.25)	0.54 (0.15, 1.90)	0.42 (0.08, 2.24)	0.23 (0.04, 1.45)
8.73 (0.99, 102.50)	7.49 (0.97, 76.77)	7.22 (1.68, 33.76)	4.42 (0.99, 22.11)	3.89 (0.58, 26.52)	2.86 (0.63, 17.17)	2.87 (0.69, 12.49)	2.74 (0.64, 11.61)	2.33 (1.19, 4.74)	2.26 (1.01, 5.05)	2.23 (0.99, 5.25)	1.58 (0.90, 3.29)	1.29 (0.23, 6.74)	A	0.85 (0.37, 1.99)	0.68 (0.08, 5.43)	0.55 (0.31, 0.87)	0.29 (0.13, 0.64)
10.18 (1.02, 122.95)	8.84 (0.98, 102.46)	8.50 (1.63, 46.14)	5.13 (0.94, 31.55)	4.57 (0.59, 35.41)	3.36 (0.60, 24.07)	3.37 (0.65, 17.90)	3.23 (0.63, 16.10)	2.72 (0.97, 7.99)	2.64 (0.82, 8.43)	2.62 (0.87, 8.02)	1.84 (0.83, 4.93)	1.49 (0.24, 9.27)	1.17 (0.50, 2.70)	B2	0.80 (0.08, 7.36)	0.64 (0.25, 1.42)	0.35 (0.11, 1.02)
12.63 (0.63, 326.42)	11.26 (0.59, 248.08)	10.51 (1.09, 110.19)	6.53 (0.49, 92.87)	5.74 (0.74, 46.76)	4.24 (0.31, 69.95)	4.21 (0.33, 55.38)	4.05 (0.41, 39.93)	3.43 (0.38, 32.90)	3.28 (0.35, 33.26)	3.25 (0.48, 23.93)	2.33 (0.28, 22.62)	1.87 (0.53, 6.80)	1.47 (0.18, 12.61)	1.25 (0.14, 11.97)	A+F	0.80 (0.10, 6.43)	0.43 (0.05, 3.97)
16.15 (1.80, 193.81)	13.94 (1.77, 150.85)	13.30 (3.27, 62.63)	8.13 (1.76, 44.40)	7.15 (1.11, 49.45)	5.29 (1.09, 34.34)	5.31 (1.19, 25.59)	5.08 (1.24, 21.53)	4.29 (2.02, 10.10)	4.14 (1.66, 11.34)	4.11 (1.96, 9.62)	2.88 (1.58, 6.95)	2.36 (0.45, 12.53)	1.83 (1.15, 3.21)	1.55 (0.71, 3.95)	1.25 (0.16, 10.20)	H	0.54 (0.24, 1.28)
29.95 (3.14, 386.68)	25.61 (3.06, 289.80)	24.69 (4.94, 136.39)	15.21 (2.99, 85.45)	13.32 (1.73, 105.48)	9.85 (1.77, 68.84)	9.78 (1.95, 53.25)	9.45 (1.90, 47.73)	8.00 (3.22, 21.45)	7.74 (2.50, 24.41)	7.66 (2.65, 23.56)	5.38 (2.16, 16.38)	4.40 (0.69, 26.80)	3.41 (1.56, 7.78)	2.89 (0.98, 9.24)	2.32 (0.25, 21.35)	1.86 (0.78, 4.23)	B1

Figure S8

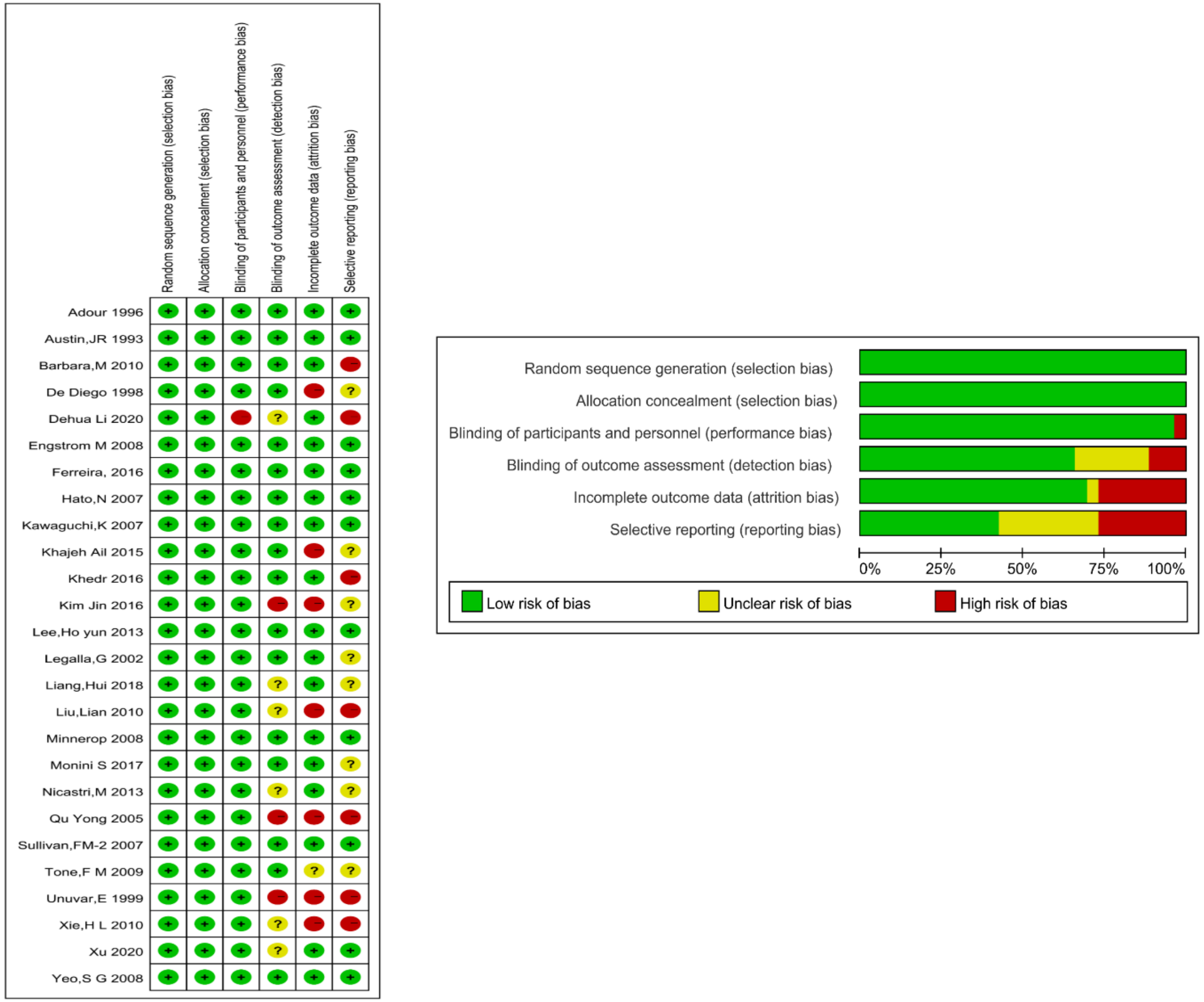
Random sequence generation (selection bias)
Allocation concealment (selection bias)
Blinding of participants and personnel (performance bias)
Blinding of outcome assessment (detection bias)
Incomplete outcome data (attrition bias)
Selective reporting (reporting bias)
Adour 1996
Austin,JR 1993
Barbara,M 2010
De Diego 1998
Dehua Li 2020
Engstrom M 2008
Ferreira, 2016
Hato,N 2007
Kawaguchi,K 2007
Khajeh Ail 2015
Khedr 2016
Kim Jin 2016
Lee,Ho yun 2013
Legalla,G 2002
Liang,Hui 2018
Liu,Lian 2010
Minnerop 2008
Monini S 2017
Nicastri,M 2013
Qu Yong 2005
Sullivan,FM-2 2007
Tone,F M 2009
Unuvar,E 1999
Xie,H L 2010
Xu 2020
Yeo,S G 2008
0%
25%
50%
75%
100%
Low risk of bias
Unclear risk of bias
High risk of bias

[1]. Barbara M, Antonini G, Vestri A, Volpini L, Monini S. Role of Kabat physical rehabilitation in Bell's palsy: a randomized trial. Acta Otolaryngol. 2010;130(1):167-72. Epub 2009/05/12. doi: 10.3109/00016480902882469. PubMed PMID: 19430987.
[2]. Engström M, Berg T, Stjernquist-Desatnik A, Axelsson S, Pitkäranta A, Hultcrantz M, et al. Prednisolone and valaciclovir in Bell's palsy: a randomised, double-blind, placebo-controlled, multicentre trial. The Lancet Neurology. 2008;7(11):993-1000. doi: 10.1016/s1474-4422(08)70221-7.
[3]. Khedr EM, Badry R, Ali AM, Abo El-Fetoh N, El-Hammady DH, Ghandour AM, et al. Steroid/Antiviral for the treatment of Bell's palsy: Double blind randomized clinical trial. Restor Neurol Neurosci. 2016;34(6):897-905. Epub 2016/10/01. doi: 10.3233/RNN-150605. PubMed PMID: 27689547.
[4]. Kim J, Choi JY. The effect of subthreshold continuous electrical stimulation on the facial function of patients with Bell's palsy. Acta Otolaryngol. 2016;136(1):100-5. Epub 2015/09/25. doi: 10.3109/00016489.2015.1083121. PubMed PMID: 26399994.
[5]. Liang H, Li Z, Lin H, Chen J. [Effects of methycobal iontophoresis combined with balance acupuncture on peripheral facial paralysis]. Zhongguo zhen jiu = Chinese acupuncture & moxibustion. 2018;38(9):955-60. Epub 2019/01/24. doi: 10.13703/j.0255-2930.2018.09.013. PubMed PMID: 30672181.
[6]. Monini S, Buffoni A, Romeo M, Di Traglia M, Filippi C, Atturo F, et al. Kabat rehabilitation for Bell's palsy in the elderly. Acta Otolaryngol. 2017;137(6):646-50. Epub 2016/12/15. doi: 10.1080/00016489.2016.1262553. PubMed PMID: 27967288.
[7]. Nicastri M, Mancini P, De Seta D, Bertoli G, Prosperini L, Toni D, et al. Efficacy of early physical therapy in severe Bell's palsy: a randomized controlled trial. Neurorehabil Neural Repair. 2013;27(6):542-51. Epub 2013/04/04. doi: 10.1177/1545968313481280. PubMed PMID: 23549520.
[8]. Hernandez RA, Sullivan F, Donnan P, Swan I, Vale L, Group BT. Economic evaluation of early administration of prednisolone and/or aciclovir for the treatment of Bell's palsy. Fam Pract. 2009;26(2):137-44. Epub 2009/02/27. doi: 10.1093/fampra/cmn107. PubMed PMID: 19244470.
[9]. Tong FM, Chow SK, Chan PY, Wong AK, Wan SS, Ng RK, et al. A prospective randomised controlled study on efficacies of acupuncture and steroid in treatment of idiopathic peripheral facial paralysis. Acupunct Med. 2009;27(4):169-73. Epub 2009/11/28. doi: 10.1136/aim.2009.000638. PubMed PMID: 19942723.
[10]. Yeo SG, Lee YC, Park DC, Cha CI. Acyclovir plus steroid vs steroid alone in the treatment of Bell's palsy. Am J Otolaryngol. 2008;29(3):163-6. Epub 2008/04/29. doi: 10.1016/j.amjoto.2007.05.001. PubMed PMID: 18439948.
[11]. Minnerop M, Herbst M, Fimmers R, Kaabar P, Matz B, Klockgether T, et al. Bell's palsy: combined treatment of famciclovir and prednisone is superior to prednisone alone. Journal of neurology. 2008;255(11):1726-30. Epub 2008/09/05. doi: 10.1007/s00415-008-0008-6. PubMed PMID: 18769863.
[12]. Ferreira M, Firmino MJ, Marques EA, Santos PC, Duarte JA. Are corticosteroids useful in all degrees of severity and rapid recovery of Bell's palsy? Acta Otolaryngol. 2016;136(7):736-41. Epub 2016/03/24. doi: 10.3109/00016489.2016.1154604. PubMed PMID: 27003272.
[13]. Kawaguchi K, Inamura H, Abe Y, Koshu H, Takashita E, Muraki Y, et al. Reactivation of herpes simplex virus type 1 and varicella-zoster virus and

therapeutic effects of combination therapy with prednisolone and valacyclovir in patients with Bell's palsy. The Laryngoscope. 2007;117(1):147-56. Epub 2007/01/05. doi: 10.1097/01.mlg.0000248737.65607.9e. PubMed PMID: 17202945.
[14]. Lee HY, Byun JY, Park MS, Yeo SG. Steroid-antiviral treatment improves the recovery rate in patients with severe Bell's palsy. Am J Med. 2013;126(4):336-41. Epub 2013/02/12. doi: 10.1016/j.amjmed.2012.08.020. PubMed PMID: 23394867.
[15]. Austin JR, Peskind SP, Austin SG, Rice DH. Idiopathic facial nerve paralysis: a randomized double blind controlled study of placebo versus prednisone. The Laryngoscope. 1993;103(12):1326-33. Epub 1993/12/01. doi: 10.1288/00005537-199312000-00002. PubMed PMID: 8246650.
[16]. De Diego JI, Prim MP, De Sarriá MJ, Madero R, Gavilán J. Idiopathic facial paralysis: a randomized, prospective, and controlled study using single-dose prednisone versus acyclovir three times daily. The Laryngoscope. 1998;108(4 Pt 1):573-5. Epub 1998/04/18. doi: 10.1097/00005537-199804000-00020. PubMed PMID: 9546272.
[17]. Li DH, Li J, Ye XQ, Peng Q. [Early treatment of suspension moxibustion for Bell's palsy in acute stage]. Zhongguo zhen jiu = Chinese acupuncture & moxibustion. 2020;40(2):123-8. Epub 2020/02/27. doi: 10.13703/j.0255-2930.20190101-k00014. PubMed PMID: 32100495.
[18]. Hato N, Yamada H, Kohno H, Matsumoto S, Honda N, Gyo K, et al. Valacyclovir and prednisolone treatment for Bell's palsy: a multicenter, randomized, placebo-controlled study. Otology & neurotology : official publication of the American Otological Society, American Neurotology Society [and] European Academy of Otology and Neurotology. 2007;28(3):408-13. Epub 2007/04/07. doi: 10.1097/01.mao.0000265190.29969.12. PubMed PMID: 17414047.
[19]. Lagalla G, Logullo F, Di Bella P, Provinciali L, Ceravolo MG. Influence of early high-dose steroid treatment on Bell's palsy evolution. Neurological sciences : official journal of the Italian Neurological Society and of the Italian Society of Clinical Neurophysiology. 2002;23(3):107-12. Epub 2002/10/23. doi: 10.1007/s100720200035. PubMed PMID: 12391494.
[20]. Liu LA, Zhu ZB, Qi QH, Ni SS, Cui CH, Xing D. [Comparison of therapeutic effects of peripheral facial paralysis in acute stage by different interventions]. Zhongguo zhen jiu = Chinese acupuncture & moxibustion. 2010;30(12):989-92. Epub 2011/02/05. PubMed PMID: 21290835.
[21]. Adour KK, Ruboyianes JM, Von Doersten PG, Byl FM, Trent CS, Quesenberry CP, Jr., et al. Bell's palsy treatment with acyclovir and prednisone compared with prednisone alone: a double-blind, randomized, controlled trial. The Annals of otology, rhinology, and laryngology. 1996;105(5):371-8. Epub 1996/05/01. doi: 10.1177/000348949610500508. PubMed PMID: 8651631.
[22]. Unüvar E, Oğuz F, Sidal M, Kiliç A. Corticosteroid treatment of childhood Bell's palsy. Pediatric neurology. 1999;21(5):814-6. Epub 1999/12/11. doi: 10.1016/s0887-8994(99)00099-5. PubMed PMID: 10593672.
[23]. Khajeh A, Fayyazi A, Soleimani G, Miri-Aliabad G, Shaykh Veisi S, Khajeh B. Comparison of the Efficacy of Combination Therapy of Prednisolone - Acyclovir with Prednisolone Alone in Bell's Palsy. Iranian journal of child neurology. 2015;9(2):17-20. Epub 2015/07/30. PubMed PMID: 26221158; PubMed

Central PMCID: PMCPMC4515336.
[24]. Qu Y. [Clinical observation on acupuncture by stages combined with exercise therapy for treatment of Bell palsy at acute stage]. Zhongguo zhen jiu = Chinese acupuncture & moxibustion. 2005;25(8):545-7. Epub 2005/11/29. PubMed PMID: 16309052.
[25]. Xu LW, Quan XM, Song CX, Liu YL, Xu HY. [Influence on orbicularis oculi muscle in the patients with facial neuritis treated with the penetra-ting needling at Cuanzhu (BL2) and Yuyao (EX-HN4) combined with the perpendicular needling at Shenmai (BL62)]. Zhen ci yan jiu = Acupuncture research. 2020;45(9):735-9. Epub 2020/09/23. doi: 10.13702/j.1000-0607.190894. PubMed PMID: 32959557.
[26]. Xie HL, Cao XM, Huang SZ, Chen SJ, Zhu F, Zheng XY. [Effect of shallow needling combined with acupoint application on the acute stage of peripheral facial paralysis]. Zhongguo zhen jiu = Chinese acupuncture & moxibustion. 2010;30(7):567-9. Epub 2010/09/25. PubMed PMID: 20862940.
